# A SuperLEphilic/Superhydrophobic and Thermostable Separator Based on Silicone Nanofilaments for Li Metal Batteries

**DOI:** 10.1016/j.isci.2019.06.010

**Published:** 2019-06-11

**Authors:** Yanfei Yang, Bucheng Li, Lingxiao Li, Stefan Seeger, Junping Zhang

**Affiliations:** 1 Center of Eco-material and Green Chemistry, Lanzhou Institute of Chemical Physics, Chinese Academy of Sciences, Lanzhou 730000, P.R. China; 2 Center of Materials Science and Optoelectronics Engineering, University of Chinese Academy of Sciences, Beijing 100049, China; 3 Department of Chemistry, University of Zurich, Zurich 8057, Switzerland

**Keywords:** Chemistry, Electrochemistry, Nanoelectrochemistry, Materials Science, Energy Materials

## Abstract

Conventional polyolefin separators suffer from poor wettability to liquid electrolytes (LEs). Although some modified separators exhibit improved wettability, they are hydrophilic, causing inevitable moisture uptake. Trace water could result in poor performance and safety hazard of Li metal batteries. Here, we report a design idea of superLEphilic/superhydrophobic and thermostable separators by modifying the Celgard separator using silicone nanofilaments. The separator features low moisture uptake (∼0%), fast LE diffusion (454 ms), and high LE uptake (287.8%), LE retention rate, and Li^+^ conductivity. Consequently, the Li/LiFePO_4_ cells show high cycling stability (96.05% after 350 cycles), good rate performance (125 mA h g^−1^ at 5.0 C), low resistance, and stable open circuit voltage at 160°C. Moreover, the separator could improve performance of the other Li metal batteries with high-voltage cathodes and the LiFePO_4_/graphite pouch cells. This work provides an avenue for designing advanced separators by using bioinspired superwetting surfaces.

## Highlights

• A superLEphilic/superhydrophobic separator is first reported for Li metal batteries
• The separator has low moisture uptake and could improve performance of Li/Li cells
• The separator features fast LE diffusion, high LE uptake, and Li^+^ conductivity
• The separator could enhance performance of high-voltage Li metal batteries

## Graphical Abstract

**Figure:**
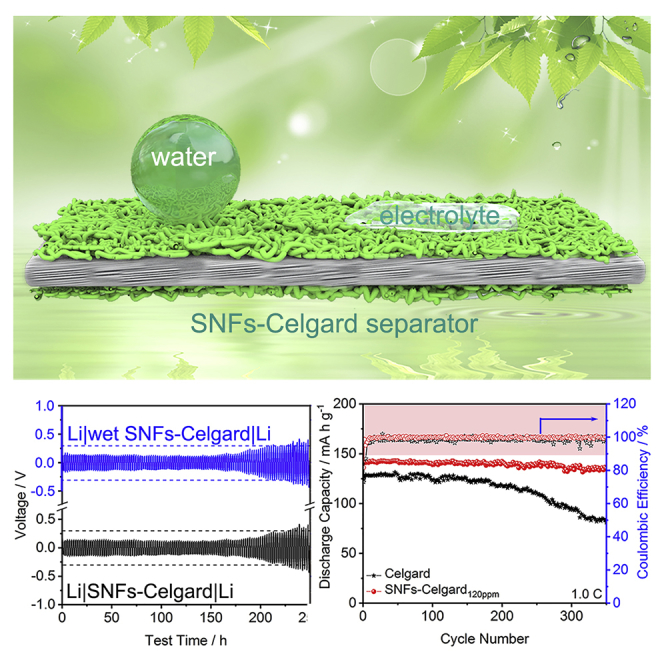

## Introduction

Li batteries are widely used, for example, in mobile communications, portable electronic devices, and automotive technology (Seh et al., 2016, Zhang et al., 2017). However, ignition and explosion accidents of Li batteries have become more frequent recently, such as the well-known Samsung Note 7 and iPhone issues, which have caused serious safety concerns (Liu et al., 2018a). Some studies suggest that these accidents are closely related to the separator (Li et al., 2017d, Liu et al., 2018a, Palacin and de Guibert, 2016). The separator plays a key role in the capacity, cycling stability, and safety of Li batteries (Liu et al., 2018b, Lu et al., 2017). Currently, microporous polyolefin membranes are widely used as separators in commercial Li batteries because of their fascinating properties (Lu et al., 2017, Zhang et al., 2017). However, polyolefin separators show poor wettability toward liquid electrolytes (LEs) and low LE uptake (Ryou et al., 2011), which lead to low Li^+^ conductivity and high internal resistance (Lee et al., 2014). Moreover, polyolefin separators exhibit inferior thermostability (Arora and Zhang, 2004, Lee et al., 2014), which may cause internal short-circuiting, ignition, and even explosion of Li batteries. These intrinsic drawbacks limit the development of advanced Li batteries (Lee et al., 2014).

To overcome these drawbacks, some efforts have been made to construct separators using materials other than polyolefins (Kim et al., 2017, Lin et al., 2016). However, these separators cannot efficiently balance all the requirements and may introduce new drawbacks, such as inferior mechanical properties (Dai et al., 2016). Considering that polyolefin separators possess many excellent properties, surface modification via coating (Dai et al., 2016, Hu et al., 2016, Ryou et al., 2011) and grafting (Abbas et al., 2017, Li et al., 2017b) is an effective approach to overcome the drawbacks. Ceramic and/or polymer coatings have been used to modify polyolefin separators (Cho et al., 2017, Jeon et al., 2016). Despite improvements in wettability and thermostability, the coatings show defects, including blocked pores, increased thickness, and reduced Li^+^ conductivity, which cause serious performance degradation of Li batteries. In contrast, surface grafting of functional groups and/or polymers could minimize the thickness increase and maintain the microporous structure (Yu et al., 2016). Although the modified polyolefin separators show improved wettability and thermostability, they are also hydrophilic (Chen et al., 2016, Dai et al., 2016, Ryou et al., 2011). Thus, moisture uptake is inevitable during the use and storage periods, which is unfavorable for the assembly and performance of Li batteries, especially for Li metal batteries. At present, Li metal anode is receiving great attention owing to its highest theoretical specific capacity (3,860 mA h g^−1^) and lowest redox potential (−3.04 V versus standard hydrogen electrode) (Cha et al., 2018, Fan et al., 2018, Liu et al., 2017). However, the uncontrollable dendrite Li growth in Li metal batteries caused many issues, such as low Coulombic efficiency, poor cycling stability, and safety hazard, which are the huge barriers to their real-world applications (Li et al., 2018a, Li et al., 2018c, Xu et al., 2018). In fact, trace water could be involved in side reactions at the interface of electrodes (Li et al., 2018c). For example, even if there is trace water in Li metal batteries, the exothermal reactions between Li metal and water will not only induce consumption of Li anode and LEs but also accelerate dendrite Li growth, resulting in poor performance and serious safety hazard of Li metal batteries. Furthermore, the exothermal reactions may trigger diverse safety issues of Li metal batteries, such as shrinkage of the separator, ignition of the flammable separator and LE, and even explosion of batteries.

Bioinspired superhydrophobic surfaces, characterized by extremely high water repellency, have many applications, including waterproof coatings (Erbil et al., 2003), self-cleaning surfaces (Zhang et al., 2014), and oil/water separation (Li et al., 2017c). A nonfluorinated superhydrophobic surface is commonly oleophilic or superoleophilic, owing to the big difference in surface tension between water (72.8 mN m^−1^) and most of organic liquids (<30 mN m^−1^) (Cheng and Rodak, 2005). The surface tension of common LEs is 26.56–31.35 mN m^−1^ (Table S1). Thus, there is a great chance to prepare superLEphilic/superhydrophobic polyolefin separators for high-performance Li metal batteries. SuperLEphilic separators are defined as the separators with contact angles (CAs) of LEs close to 0°. The superLEphilicity may enhance LE uptake and retention and Li^+^ conductivity owing to fast and complete wetting of the separator by LEs and thus may improve the battery performance (Lee et al., 2014). Although some LEphilic separators have been reported, superLEphilic separators are rare (Li et al., 2017a, Shi et al., 2016). The superhydrophobicity may eliminate side reactions and safety issues of Li metal batteries by reducing moisture uptake and also avoid additional trouble in battery assembly, as the drying process of separators before battery assembly is energy- and time-consuming. To the best of our knowledge, there has been no report about superLEphilic/superhydrophobic separators to date in rechargeable batteries. It is challenging to rationally design and fabricate superLEphilic/superhydrophobic separators without sacrificing other properties of polyolefin separators.

Here, we report for the first time a design idea of superLEphilic/superhydrophobic separators for Li metal batteries. The separator is prepared by growth of silicone nanofilaments (SNFs) on the surface of a polypropylene separator (Celgard 2400) by hydrolytic condensation of trichloromethylsilane (TCMS) in toluene. The microstructure of the SNFs determines wettability, LE uptake and retention, Li^+^ conductivity, and thermostability of the SNFs-Celgard separator and can be tuned by the water concentration in toluene. The separator can be easily wetted by diverse LEs, and thus the LE uptake and retention and Li^+^ conductivity are substantially enhanced. The separator cannot be wetted by water, and the moisture uptake is extremely low. Consequently, the performance of various Li metal batteries and the LiFePO_4_/graphite pouch cells is evidently improved by the SNFs-Celgard separator.

## Results

### Preparation of SNFs-Celgard Separators

**Figure 1:**
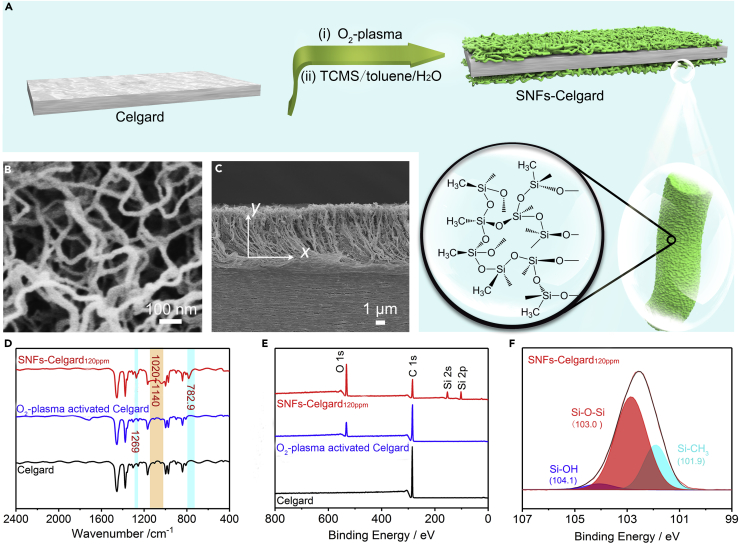
Preparation and Characterization of SNFs-Celgard Separators (A) Schematic illustration of preparation of the SNFs-Celgard separator by O_2_-plasma activation and subsequent SNFs growth, and chemical structure of the SNFs. (B and C) (B) SEM and (C) cross-sectional SEM images of the SNFs-Celgard_120ppm_ separator. See also Figures S2 and S3. (D–F) (D) FTIR spectra, (E) XPS spectra, and (F) high-resolution Si 2p spectrum of the separators. See also Figure S5 and Table S2.

Figure 1A shows the preparation of the SNFs-Celgard separator by O_2_-plasma activation and subsequent SNFs growth. O_2_-plasma was used to activate the chemically inert Celgard separator by forming reactive hydroxyl groups without evidently changing its microporous structure (Figure S1). Once immersed in the fresh TCMS/toluene solution containing a small amount of water, TCMS will hydrolyze and self-assemble into a highly porous polymeric network that is composed of a large amount of randomly deposited SNFs on the Celgard separator (Figures 1B and S2). The SNFs are 40–60 nm in diameter and several micrometers in length. The SNFs layer is 5.5 μm thick on each side (Figures 1C and S3), which seems to be thick. However, our previous studies have shown that the SNFs layer is soft and highly porous (Chu and Seeger, 2015, Meseck et al., 2014). The volume fraction of the SNFs layer is very low, 2.82% along the *y* axis (Meseck et al., 2014). Thus, the SNFs layer is compressible during battery assembly and the thickness of the SNFs layer in the coin cells should be ∼0.155 μm on each side, assuming 100% strain of the space in the SNFs layer. The SNFs have no influence on the appearance of the Celgard separator (Figure S4). Moreover, the microstructure of the SNFs is tunable simply by the water concentration in toluene. The separators were termed as SNFs-Celgard_50ppm_, SNFs-Celgard_120ppm_, and SNFs-Celgard_200ppm_ according to the water concentration in toluene.

In the Fourier transform infrared (FTIR) spectrum of the SNFs-Celgard separator (Figure 1D), the new absorption bands ascribed to Si-O-Si and Si-O-C stretching (1,020–1,140 cm^−1^) and Si-CH_3_ bending (1,269 and 782.9 cm^−1^) were observed (Ward et al., 2003). In the X-ray photoelectron spectra (XPS) of the SNFs-Celgard separator (Figure 1E), the Si 2s, Si 2p, C 1s, and O 1s peaks appeared. This is in agreement with the elemental maps of the separator (Figure S3B). The O/C/Si atomic ratio is 1/1.48/0.69 on the surface of the SNFs-Celgard_120ppm_ separator (Table S2), which is consistent with the theoretical ratio of 1/0.66/0.66 for polymethylsilsesquioxane (Zimmermann et al., 2008). The higher C content is due to organic contamination during storage of the sample at ambient conditions in the interval between preparation and XPS analysis (Zimmermann et al., 2008). The absence of Cl 1s peak indicates complete hydrolysis of TCMS on the surface of the separator. The SNFs are mainly composed of Si-O-Si and Si-C bonds with a few Si-OH groups as demonstrated by the Si 2p spectrum of the SNFs-Celgard separator (Figure 1F) (Nozawa and Aramaki, 1999). According to the FTIR and XPS analyses, and the hydrolytic condensation of TCMS in toluene (Figure S5) (Gao and McCarthy, 2006, Zhang and Seeger, 2011b), the chemical structure of the SNFs is shown in Figure 1A.

### SuperLEphilicity of SNFs-Celgard Separators

**Figure 2:**
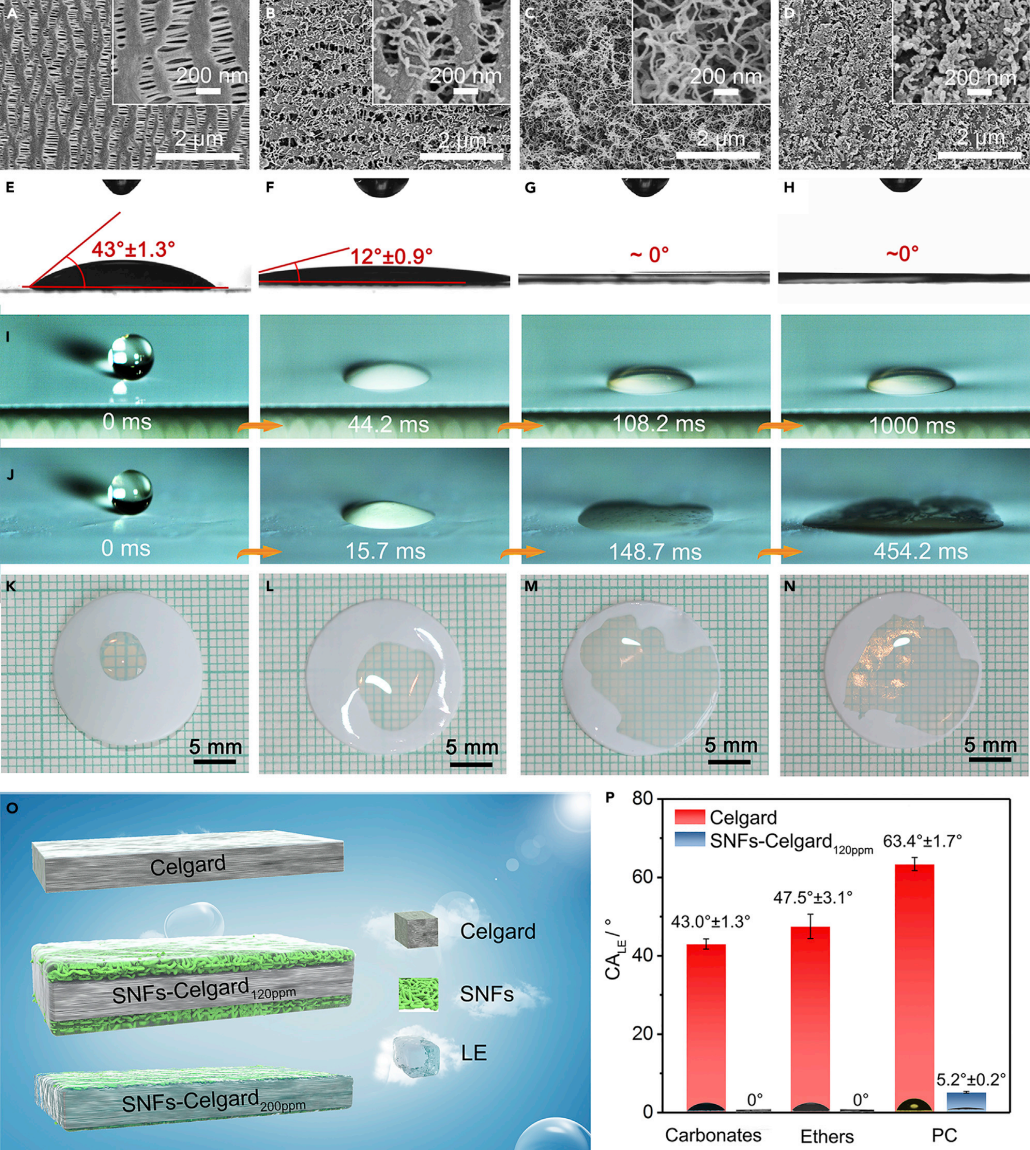
SuperLEphilicity of SNFs-Celgard Separators (A–H) SEM images and CA_LE_ images of the (A and E) Celgard, (B and F) SNFs-Celgard_50ppm_, (C and G) SNFs-Celgard_120ppm_, and (D and H) SNFs-Celgard_200ppm_ separators. (I and J) Dynamic wetting process of the (I) Celgard and (J) SNFs-Celgard_120ppm_ separators by the LE droplets (6 μL) released from a height of 5 mm. See also Videos S1 and S2. (K–N) Photographs of the LE droplets (10 μL) after they were dropped onto the (K) Celgard, (L) SNFs-Celgard_50ppm_, (M) SNFs-Celgard_120ppm_, and (N) SNFs-Celgard_200ppm_ separators for 10 s. See also Figures S7 and S8. (O) Schematic illustrations of the separators with absorbed LE. (P) CA_LE_ of the LEs with different surface tensions on the surface of the Celgard and SNFs-Celgard_120ppm_ separators (shown as means ± SD, n = 6). Carbonates: 1 M LiPF_6_ in 1:1 (v/v) EC and DMC. Ethers: 1 M LiTFSI and 0.2 M LiNO_3_ in 1:1 (v/v) 1,2-dimethoxyethane and 1,3-dioxacyclopentane. PC: 1 M LiPF_6_ in propylene carbonate.

The Celgard separator is microporous with a pore diameter of several hundreds of nanometers (Figure 2A). The Celgard separator shows poor wettability toward the LE (CA_LE_ = 43.0°), which not only reduces the power density and cycle life of Li batteries but also makes the Li battery assembly time-consuming, as the LE filling is the slowest step in Li battery assembly (Arora and Zhang, 2004, Dai et al., 2016). O_2_-plasma activation could make the Celgard separator LEphilic and hydrophilic. However, the CA_water_ gradually increased with storage time (Figure S6), owing to spontaneous thermal motion of the polyolefin molecular chains to minimize the surface energy (Li and Zhang, 2016). After modification with TCMS at a low water concentration of 50 ppm, only sparse and short SNFs were grown on the Celgard separator (Figure 2B), because the hydrolytic condensation of TCMS could not proceed sufficiently. According to the Cassie-Baxter and the Wenzel models (Cassie and Baxter, 1944, Wenzel, 1936), introduction of a proper microstructure could make an LEphilic surface more LEphilic or even superLEphilic owing to the capillary effect (Zhang and Seeger, 2011a). Thus, the SNFs-Celgard_50ppm_ separator has a smaller CA_LE_ of 12° compared with the Celgard separator (Figures 2E and 2F). Thick and long SNFs were formed upon increasing the water concentration to 120 ppm (Figure 2C), because the “vertical polymerization” of TCMS was promoted (Fadeev and McCarthy, 2000). Meanwhile, the CA_LE_ decreased to ∼0° (Figure 2G), indicating formation of the superLEphilic separator. This is because the SNFs loosely stacked together and formed a 3D cross-linked polymeric network with high surface area (Meseck et al., 2014). This is further confirmed by the higher porosity of the SNFs-Celgard_120ppm_ separator (51.9%) than the others (Table S3). With further increasing the water concentration to 200 ppm, a dense layer of short and worm-like SNFs was formed (Figure 2D), because the reaction was too violent. This is consistent with previous studies (Gao and McCarthy, 2006, Zhang and Seeger, 2011b). The SNFs formed at a water concentration of 200 ppm are still sufficient to make the separator superLEphilic (Figure 2H).

The dynamic wetting process of the SNFs-Celgard separators by the LE was observed using a high-speed digital camera with the Celgard separator for comparison. When dropped from a height of 5 mm onto the Celgard separator for 108.2 ms, the LE droplet reached its equilibrium state (Figure 2I and Video S1). No shape change and diffusion of the LE droplet were observed with further increasing the contact time to 1 s. In contrast, the LE droplet wetted and completely diffused into the entire SNFs-Celgard_120ppm_ separator in ∼454.2 ms (Figure 2J and Videos S1 and S2), owing to its superLEphilicity. This is faster than all the reported separators, including the recently reported HAP/CF separator, into which the LE droplet penetrated in 5 s (Li et al., 2017a). For the SNFs-Celgard_50ppm_ and SNFs-Celgard_200ppm_ separators, the LE droplets have slower diffusion speed and smaller wetting areas compared with the SNFs-Celgard_120ppm_ separator (Video S1). This is because the SNFs are sparse and short. After 10 s, the LE wetting areas for the SNFs-Celgard_120ppm_ and SNFs-Celgard_200ppm_ separators are 1.75 and 1.61 cm^2^, respectively, which are much larger than that of the SNFs-Celgard_50ppm_ (0.51 cm^2^) and Celgard (0.12 cm^2^) separators (Figures 2K–2N). The LE filling is the slowest step in the assembly of Li batteries with the Celgard separator. Thanks to the superLEphilicity, the cell with the SNFs-Celgard_120ppm_ separator can be assembled very quickly. During cell assembly, 50 μL of the LE was dripped on the surface of the separator, and the time when the separator was completely wetted was recorded. The Celgard separator was completely wetted in ∼39 min, which is about 62 times more than that of the SNFs-Celgard_120ppm_ separator (∼37 s).

Video S1. Impacting of 6 μL LE Droplets on the Celgard and SNFs-Celgard Separators, Related to Figure 2 This video highlights superLEphilicity of the SNFs-Celgard_120ppm_ separator. The video was recorded at 4,000 fps using a high-speed digital camera (FASTCAM Mini UX100, Photron, Japan). Still images are shown in Figures 2I and 2J.

Video S2. Impacting of 6 μL LE Droplets on the Celgard and SNFs-Celgard_120ppm_ Separators, Related to Figure 2 This video highlights that the Celgard film and the SNFs layer of the SNFs-Celgard_120ppm_ separator are quickly wetted by LE simultaneously. The video was recorded at 4,000 fps using a high-speed digital camera (FASTCAM Mini UX100, Photron, Japan).

The SNFs are beneficial to enhance the LE uptake (Table S3). The LE uptake of the SNFs-Celgard_120ppm_ separator is 287.8%, which is much higher than that of the SNFs-Celgard_200ppm_ (196.9%), SNFs-Celgard_50ppm_ (165.5%), and Celgard (91.3%) separators and the previously reported separators (80%–253%) (Dai et al., 2016, Li et al., 2017a, Li et al., 2017b, Ryou et al., 2011). This is because the 3D cross-linked polymeric network of the SNFs provides a large space to accommodate the LE (Figures 2O and S3). Besides high LE uptake, the SNFs-Celgard_120ppm_ separator shows high LE retention rate owing to the high affinity between the LE and the separator. After storage in room conditions for 30 min, the LE retention rate of the SNFs-Celgard_120ppm_ separator is 85.6%, which is higher than that of the SNFs-Celgard_200ppm_ (76.2%), SNFs-Celgard_50ppm_ (67.0%), and Celgard (44.2%) separators (Figure S7). After 1 h, the Celgard separator became half dry, whereas the SNFs-Celgard_120ppm_ separator was still completely wetted by the LE, demonstrating a high LE retention rate (Figure S8). It is well known that the Li^+^ conductivity of a separator is closely related to the LE uptake and retention rate (Dai et al., 2016). The Li^+^ conductivity of the SNFs-Celgard_120ppm_ separator is 1.02 mS cm^−1^, which is higher than that of the SNFs-Clegard_200ppm_ (0.832 mS cm^−1^), SNFs-Celgard_50ppm_ (0.740 mS cm^−1^), and Celgard (0.727 mS cm^−1^) separators (Figure S9 and Table S3). The Li^+^ conductivity was increased by 40% by introducing SNFs onto the Celgard separator. Moreover, The Si-O groups of SNFs can act as Lewis acid to trap a sufficient amount of Li salt anions (Lee et al., 2007, Wang et al., 2015), thus increasing the dissociation degree of Li salt. Consequently, the SNFs-Celgard_120ppm_ separator showed higher Li^+^ diffusion than the Celgard separator. To support this view, the *t*_Li_ was measured (Figure S10). The *t*_Li_ is 0.43 for the Celgard separator, which is similar to the reported data (He et al., 2018). However, the *t*_Li_ was significantly enhanced to 0.59 for the SNFs-Celgard_120ppm_ separator.

To further demonstrate superLEphilicity of the SNFs-Celgard_120ppm_ separator, three commonly used LEs with different surface tensions were studied, e.g., carbonates (27.79 mN m^−1^), ethers (26.56 mN m^−1^), and PC (31.35 mN m^−1^) (Table S1). The Celgard separator showed CA_LE_ of 43.0° (carbonates), 47.5° (ethers), and 63.4° (PC), which are similar to the reported data (Hao et al., 2016, Lee et al., 2015). The SNFs-Celgard_120ppm_ separator showed significantly different wettability towards these LEs (Figure 2P). The SNFs-Celgard_120ppm_ separator showed CA_LE_ of ∼0° for both carbonates- and ethers-based LEs, and CA_LE_ of 5.2° for the strong polar PC-based LE. After 10 s, the wetting areas of the carbonates-, ethers-, and PC-based LEs on the SNFs-Celgard_120ppm_ separator are 1.75, 1.43, and 1.13 cm^2^, respectively, which are much higher than those of the Celgard separator (Figure S11).

### Superhydrophobicity of SNFs-Celgard Separators

**Figure 3:**
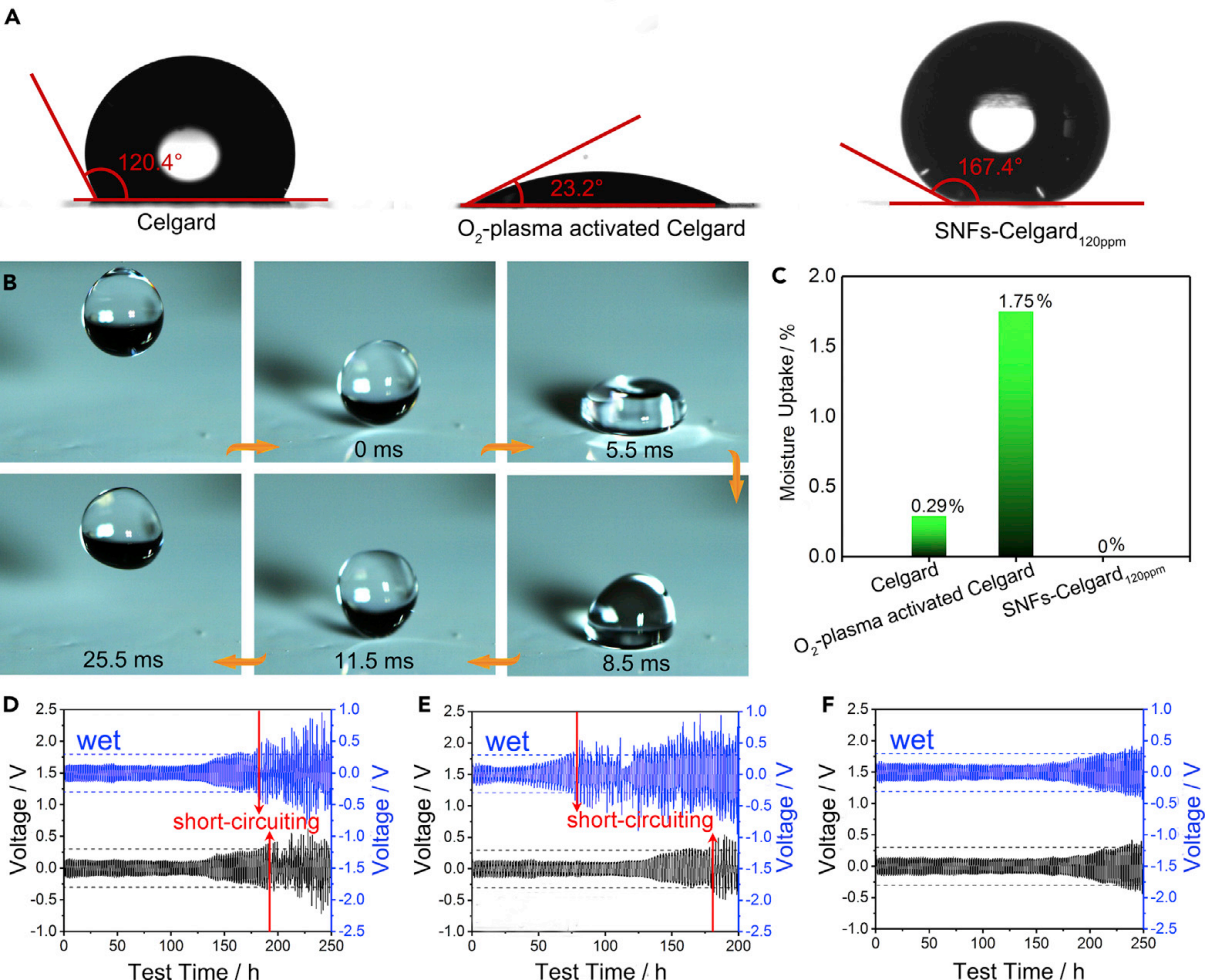
Superhydrophobicity of SNFs-Celgard Separators (A) CA_water_ images of different separators. (B) Dynamic wetting process of the SNFs-Celgard_120ppm_ separator by water. See also Video S3. (C) Moisture uptake of the separators. (D–F) Voltage-time curves of the Li symmetric cells with the (D) Celgard, (E) O_2_-plasma-activated Celgard, and (F) SNFs-Celgard_120ppm_ separators. The amount of plated Li is 1.0 mA h cm^−2^, and the current density is 1.0 mA cm^−2^ in each cycle. See also Figures S14 and S15.

Wettability of the separators towards water is shown in Figures 3A and S12. The CA_water_ is 120.4° for the Celgard separator. The CA_water_ is 23.2° for the O_2_-plasma activated Celgard, as hydroxyl groups were generated on the surface of the separator. The SNFs-Celgard_50ppm_ separator is close to superhydrophobic with a CA_water_ of 149.5° and a water sliding angle of 42.3°. With increasing the water concentration, the SNFs-Celgard separators became superhydrophobic. The SNFs-Celgard_120ppm_ has a CA_water_ of 167.4°, and the water droplets could easily roll off the 1° tilted separator. The SNFs-Celgard_200ppm_ separator has slightly lower superhydrophobicity, as the SNFs are shorter. According to the Cassie-Baxter and the Wenzel models (Cassie and Baxter, 1944, Wenzel, 1936), introduction of a proper microstructure could make a hydrophobic surface more hydrophobic or even superhydrophobic. In addition, the methyl groups on the surface of the SNFs could decrease the surface energy of the separator, which also contributes to superhydrophobicity of the SNFs-Celgard separators. The dynamic wetting process of the SNFs-Celgard_120ppm_ separator by water is shown in Figure 3B and Video S3. The 6-μL water droplet released from a height of 5 mm bounced ∼14 times before settling down on the separator. The water droplet exhibited complete rebounds with a liquid-solid contact time of ∼11.5 ms. These results demonstrate high superhydrophobicity of the SNFs-Celgard_120ppm_ separator.

Video S3. Impacting of a 6 μL Water Droplet on the SNFs-Celgard_120ppm_ Separator, Related to Figure 3 This video highlights superhydrophobicity of the SNFs-Celgard_120ppm_ separator. The video was recorded at 4,000 fps using a high-speed digital camera (FASTCAM Mini UX100, Photron, Japan). Still images are shown in Figure 3B.

The superhydrophobicity could avoid wetting of the separator by water and may also reduce moisture uptake during the use and storage periods. This is helpful to reduce the side effects of trace water in conventional Celgard separators on the performance of Li metal batteries and additional troubles in Li metal battery assembly. To verify our hypothesis, the moisture uptake of the separators was measured by keeping the separators in a high-humidity environment (relative humidity = 92.6%, 25°C) for 7 days (Figure S13). The moisture uptake of the SNFs-Celgard_120ppm_ separator is ∼0% (Figure 3C). In contrast, the moisture uptake is 0.29% for the Celgard separator and is 1.75% for the O_2_-plasma activated Celgard separator.

To gain more insight into the effects of the superhydrophobicity and the trace water in separators on performance of Li metal batteries, the separators after moisture uptake test (termed as wet separators) were immediately used for assembly of Li symmetric cells. In Li symmetric cells, trace water could be involved in reactions at the interface of the Li anode (Li et al., 2018c), leading to fluctuations of the voltage-time curves. For the hydrophobic Celgard separator, the polarization voltage of the cell started to increase after 135 h (Figure 3D) because of fast dendrite Li growth (Liu et al., 2017). Subsequently, a sudden drop in the polarization voltage was observed after 190 h, suggesting short-circuiting in the cell due to serious dendrite Li growth (Figure S14A). The voltage-time curve of the cell with the wet Celgard separator is stable at the beginning, but the sudden drop in the polarization voltage happened earlier (after 164 h, Figure 3D). This is because the trace water in the wet separator involved in exothermal side reactions at the interface of Li anode. These side reactions not only caused inhomogeneous solid electrolyte interphase (SEI)- formation but also accelerated dendrite Li growth and Li anode pulverization (Figure S14) and ultimately resulted in earlier failure of the cell. For the hydrophilic O_2_-plasma activated separator, the voltage-time curve of the cell with the wet separator is very unstable compared with that with the normal one (Figure 3E). Also, a sudden drop in the polarization voltage, i.e., short-circuiting, happened after only 76 h. These phenomena are due to the high moisture uptake of the separator. In contrast, the cell with the SNFs-Celgard_120ppm_ separator exhibits a very stable voltage-time curve in 208 h and slow increase in the voltage in subsequent cycles (Figure 3F). No short-circuiting was observed in 250 h, indicating higher stability and safety compared with the cell with the Celgard separator. Also, different from the wet Celgard and O_2_-plasma-activated Celgard separators, the voltage-time curve of the cell with the wet SNFs-Celgard_120ppm_ separator is very stable and is similar to that with the SNFs-Celgard_120ppm_ separator (Figures 3D–3F). No dendrite Li and Li anode pulverization were observed on the surface of the cycled Li anode in the cells with the SNFs-Celgard_120ppm_ separator or the wet one (Figure S15) owing to its superhydrophobicity. Thus, there is no need to dry the SNFs-Celgard_120ppm_ separator before Li metal battery assembly, which is necessary for conventional separators. This is because the superhydrophobicity of the SNFs-Celgard_120ppm_ separator can efficiently reduce moisture uptake and then avoid the side reactions at the interface of Li anode. The above-mentioned results indicate that the superhydrophobic SNFs-Celgard_120ppm_ separator can improve performance of the Li metal battery and make battery assembly simpler.

Notably, the wettability of the SNFs-Celgard_120ppm_ separator is significantly different from all the previously reported separators (Table S4). These separators are LEphilic, and only a few of them are superLEphilic, e.g., Al_2_O_3_/PI-coated PE separator (Shi et al., 2016), HAP/CF separator (Li et al., 2017a), and commercial ceramic-coated separators (Table S4). Meanwhile, all the separators can be easily wetted by water, which means high moisture uptake. However, the SNFs-Celgard_120ppm_ separator is superLEphilic and superhydrophobic simultaneously.

It also can be concluded that the superLEphilicity, LE uptake and retention rate, Li^+^ conductivity, and superhydrophobicity of the separators are closely related to the microstructure of the SNFs, which can be controlled simply by the water concentration in toluene during hydrolysis of TCMS.

### Electrochemical Performance of Cells with SNFs-Celgard Separators

**Figure 4:**
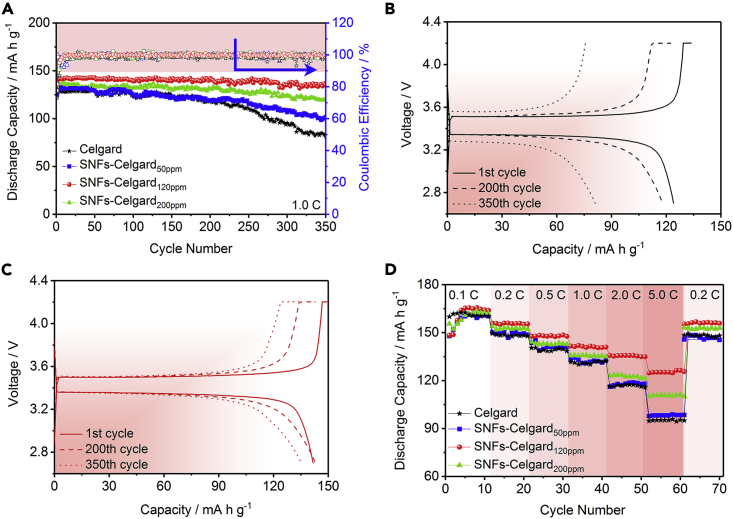
Electrochemical Performance of Cells with SNFs-Celgard Separators (A) Cycling stability of the Li/LiFePO_4_ cells with different separators. (B and C) Discharge/charge curves of the Li/LiFePO_4_ cells with the (B) Celgard and (C) SNFs-Celgard_120ppm_ separators. See also Figure S16. (D) Rate performance of the Li/LiFePO_4_ cells with different separators (1 C = 160 mA h g^−1^).

The influence of the SNFs-Celgard separators on electrochemical performance of Li metal batteries was investigated using the Li/LiFePO_4_ cells (Figure 4). The cycling stability of the cells with different separators at 1.0 C is shown in Figures 4A–4C and S16. After 350 cycles, the cells with the Celgard, SNF-Celgard_50ppm_, SNFs-Celgard_120ppm_, and SNFs-Celgard_200ppm_ separators maintained 65.32%, 81.46%, 96.05%, and 89.77% of their initial capacity, respectively. The sharp capacity drop of the cell with the Celgard separator from the 220^th^ cycle is ascribed to the dendrite Li growth, Li anode pulverization, and LE consumption (He et al., 2013, Liu et al., 2017). The voltage hysteresis of the cell with the Celgard separator showed an obvious increase from the 220^th^ cycle (Figure S17), which verified LE consumption (Liu et al., 2017). Obviously, the cell with the SNFs-Celgard_120ppm_ separator has the highest cycling stability, indicating that the separator could effectively alleviate the dendrite Li growth, Li anode pulverization, and LE consumption (Figures S17 and S18) (Li et al., 2018b, Ryou et al., 2012). The SNFs-Celgard_120ppm_ separator is superLEphilic and has a high LE uptake and retention rate. This means the SNFs have a strong affinity to Li^+^, which decreased the Li^+^ concentration gradient before Li^+^ reaching the Li anode surface (Cheng et al., 2016, Zhao et al., 2018). Consequently, the Li^+^ distributed homogeneously over the entire Li anode surface during cycling, and the interfacial interaction between Li^+^ and the Li anode was improved. It is well known that the homogeneous distribution of Li^+^ is crucial to the dendrite-free Li depositing (Cheng et al., 2017). Figure S19A shows the scanning electron microscopy (SEM) image of the SNFs-Celgard_120ppm_ separator after 350 cycles. The SNFs on the cycled separator is similar to those on the new one. Also, the cycled separator is still superhydrophobic (CA_water_ = 154.3°, Figure S19B). These results indicate that the SNFs-Celgard_120ppm_ separator has high stability in the cyclic discharge/charge process. This is further supported by the good electrochemical stability of the SNFs-Celgard_120ppm_ separator (Figure S20). This is because the Si-O groups of SNFs can act as Lewis acid to trap a sufficient amount of Li salt anions, thus delaying the irreversible oxidative decomposition of Li salt anions (Lee et al., 2007, Qiu et al., 2004, Wang et al., 2015). The gradual capacity decay of the cells with the SNFs-Celgard_50ppm_ and SNFs-Celgard_200ppm_ separators is because the spare or short SNFs cannot efficiently alleviate the dendrite Li growth and Li anode pulverization.

Figure 4D shows the rate performance of the Li/LiFePO_4_ cells with different separators. At low discharge rates, e.g., 0.1 C and 0.2 C, the Li^+^ conductivities of all the separators are enough to support the discharge rates and the cells displayed very similar rate performance (Dai et al., 2016). However, the rate performance of the cells at high discharge rates is different from each other. The cell with the SNFs-Celgard_120ppm_ separator has the lowest capacity loss upon cycling. At 5.0 C, the capacity is 125 mA h g^−1^, which is 76.25% of the capacity at 0.1 C. Instead, the capacity retentions of the cells with the Celgard, SNFs-Celgard_50ppm_, and SNFs-Celgard_200ppm_ separators are 59.28%, 61.03%, and 68.21%, respectively. At high rates, the Li^+^ conductivity and resistance act as the key factors determining the rate performance (Arora and Zhang, 2004, Dai et al., 2016). Thus, the better rate performance of the cell with the SNFs-Celgard_120ppm_ separator is attributed to the higher Li^+^ conductivity (Figures S10 and S11) and lower resistance (Figure S21).

The SNFs-Celgard_120ppm_ separator can also be applied in the Li metal batteries with high-voltage cathode materials, such as 4.9 V LiNi_0.5_Mn_1.5_O_4_ and 4.8 V Li_1.2_Mn_0.54_Ni_0.3_Co_0.3_O_2_. Compared with the Celgard separator, the Li/LiNi_0.5_Mn_1.5_O_4_ and Li/Li_1.2_Mn_0.54_Ni_0.3_Co_0.3_O_2_ cells with the SNFs-Celgard_120ppm_ separator showed higher stability in the discharge/charge process (Figures S22A and S23A), which is due to good electrochemical stability and unique wettability of the separator. Moreover, owing to higher Li^+^ conductivity of the SNFs-Celgard_120ppm_ separator and lower resistance of the corresponding cells (Figures S22B, S22C, S23B, and S23C), the cells showed better rate performance (Figures S22D and S23D). For the Li/LiNi_0.5_Mn_1.5_O_4_ cell, the capacity decay is only 30.4% with increasing the rate from 0.1 to 1.0 C, whereas that of the cell with the Celgard separator is 37.2%. For the Li/Li_1.2_Mn_0.54_Ni_0.3_Co_0.3_O_2_ cell, the value is 34.4% for the SNFs-Celgard_120ppm_ separator and 46.3% for the Celgard separator.

To further demonstrate advantages of the SNFs-Celgard_120ppm_ separator, the electrochemical performance of the Li/LiFePO_4_ cells with the SNFs-Celgard_120ppm_ separator or commercial ceramic-coated separators was compared (Figure S24). All of the separators were directly used for cell assembly. Obviously, the cycling stability and Coulombic efficiency of the cell with the SNFs-Celgard_120ppm_ separator are higher and more stable than those of the cells with the ceramic-coated separators (Figure S24A). This is because the ceramic-coated separators are hydrophilic or even superhydrophilic (Figure S24B) and the water in the separators was involved in side reactions at the interface of Li anode. All of the cells showed similar interfacial resistance before cycling (Figure S24C); however, the interfacial resistance of the cells with the ceramic-coated separators increased significantly after 100 cycles (Figure S24D). This is because of exfoliation of the ceramic layer from the polyolefin membranes during cell assembly and discharge/charge, as there is no interaction between them (Lee et al., 2014).

### Thermostability of SNFs-Celgard Separators

**Figure 5:**
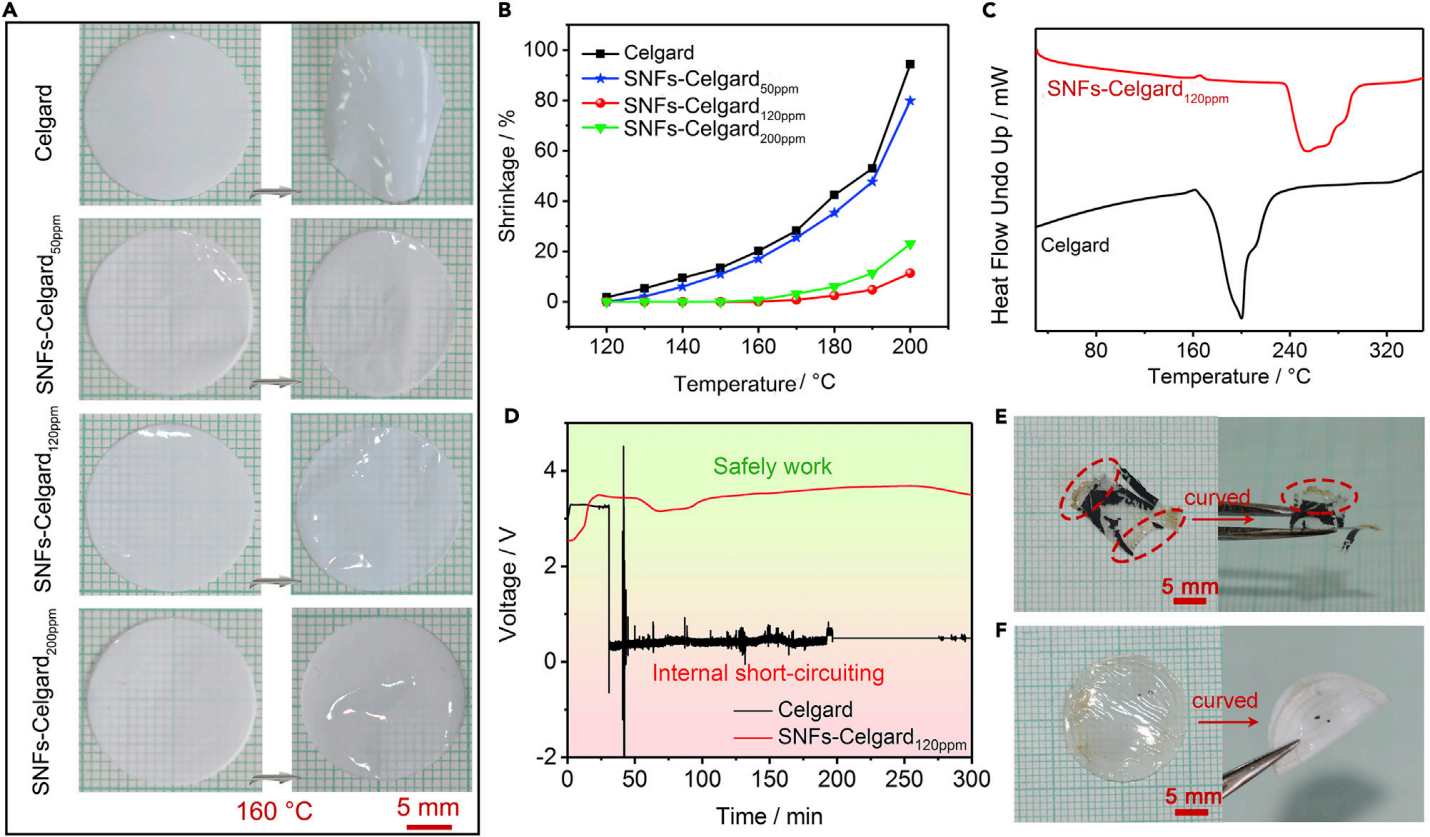
Thermostability of SNFs-Celgard Separators (A) Dimensions of the separators before and after being subjected to heat treatment at 160°C for 1 h. See also Figure S25. (B) Thermal shrinkage of the separators as a function of temperature (1 h). (C) DSC curves of the separators. See also Figure S26. (D) OCV curves of the Li/LiFePO_4_ cells with different separators at 160°C. (E and F) Photographs of the (E) Celgard and (F) SNFs-Celgard_120ppm_ separators after the OCV test.

The Celgard separator has poor thermostability owing to the low melting point (Figures 5A and S25) (Arora and Zhang, 2004, Lee et al., 2014), which may cause safety issues of Li batteries (Liu et al., 2018a). Therefore, we studied the influence of the SNFs on thermostability of the separator. At temperatures above 120°C, the thermal shrinkage of the Celgard separator is evident. The sparse and short SNFs on the surface of the SNFs-Celgard_50ppm_ separator did not improve the thermostability. The shrinkage of the SNFs-Celgard_50ppm_ separator is similar to that of the Celgard separator at the same temperature (Figure 5B). The very thick and long SNFs on the SNFs-Celgard_120ppm_ separator substantially enhanced the thermostability. The SNFs-Celgard_120ppm_ separator has no visible shrinkage at temperature up to 180°C (Figures 5B and S25). However, the SNFs-Celgard_200ppm_ separator starts to shrink at 170°C and has a shrinkage of 6% at 180°C, as the SNFs are very short. The high thermostability of the SNFs-Celgard_120ppm_ separator was further confirmed by differential scanning calorimetry (DSC) and thermal gravimetric analysis (TGA). The endothermic peak of the SNFs-Celgard_120ppm_ separator appeared at 234°C–297°C, much higher than that of the Celgard separator (160°C–220°C, Figure 5C). The evident weight loss of the Celgard separator started at 178°C, whereas that of the SNFs-Celgard_120ppm_ separator started at 215°C (Figure S26). This is because the thermostable SNFs layer functioned as the thermal resistant layer to protect the Celgard separator from shrinking, like the previously reported inorganic or polymer coatings (Dai et al., 2016, Ryou et al., 2012, Yang and Zhang, 2018). Moreover, a part of TCMS molecules diffused into micropores of the Celgard separator and deposited on the wall of the micropores in the hydrolytic condensation process, forming a composite separator, which was confirmed by the cross-section elemental maps of the SNFs-Celgard_120ppm_ separator (Figure S3B). This also contributes to the enhanced thermostability. It is worth noting that the Celgard separator was completely decomposed at 500°C in O_2_ atmosphere, but there was ∼2.40% residual SiO_2_ for the SNFs-Celgard_120ppm_ separator. This means the weight of the SNFs on the SNFs-Celgard_120ppm_ separator is 2.68%, i.e., 0.04 mg cm^−2^. Thus, the SNFs have negligible influence on the energy density of Li batteries.

To study the effect of the SNFs-Celgard_120ppm_ separator on the safety of Li metal batteries, the open circuit voltage (OCV) curves of the Li/LiFePO_4_ cells were recorded at 160°C (Figure 5D). If there is serious thermal shrinkage of the separator, battery internal short-circuiting will result in a sudden OCV drop. For the cell with the Celgard separator, the OCV dropped sharply to 0 V after 31.5 min. However, for the cell with the SNFs-Celgard_120ppm_ separator, the OCV is stable throughout the test (300 min). After the OCV test, the Celgard separator almost completely melted (Figure 5E). In contrast, the SNFs-Celgard_120ppm_ separator remained intact and could still be curved without any fracture (Figure 5F). The results demonstrate that the SNFs-Celgard_120ppm_ separator could evidently enhance safety of Li metal batteries.

### General Applicability of SNFs-Celgard Separators

To verify applicability of the SNFs-Celgard separator in Li ion batteries, the LiFePO_4_/graphite cells were tested. The cycling stability of the cells with different separators at 1.0 C (1.0 C = 133 mA h g^−1^) is shown in Figure S27A. The initial discharge capacity of the cell with the SNFs-Celgard_120ppm_ separator is 106 mA h g^−1^, which is higher than those with the Celgard (94 mA h g^−1^), SNFs-Celgard_50ppm_ (98 mA h g^−1^), and SNFs-Celgard_200ppm_ (102 mA h g^−1^) separators. After 350 cycles, the cells with the Celgard, SNF-Celgard_50ppm_, SNFs-Celgard_120ppm_, and SNFs-Celgard_200ppm_ separators maintained 62.55%, 71.53%, 84.9%, and 79.26% of their initial capacity, respectively. The cycling stability of the cell with the SNFs-Celgard_120ppm_ separator (94.3% after 100 cycles) is better than that of the cells with the Al_2_O_3_ ceramic-grafted separator (85.6% after 100 cycles) (Jiang et al., 2017) and the HAP/CF separator (92.3% after 100 cycles) (Li et al., 2017a). Moreover, the cell with the SNFs-Celgard_120ppm_ separator showed the best rate performance compared with the other separators (Figure S27B). At 5.0 C, the capacity of the cell with the SNFs-Celgard_120ppm_ separator is 58 mA h g^−1^, which is 46.03% of the capacity at 0.1 C. Instead, the capacity retention of the cell with the Celgard separator is only 29.66%. Moreover, the SNFs-Celgard_120ppm_ separator could evidently enhance the safety of Li ion batteries according to the OCV curves and the photographs of the separators after the OCV test (Figure S28).

**Figure 6:**
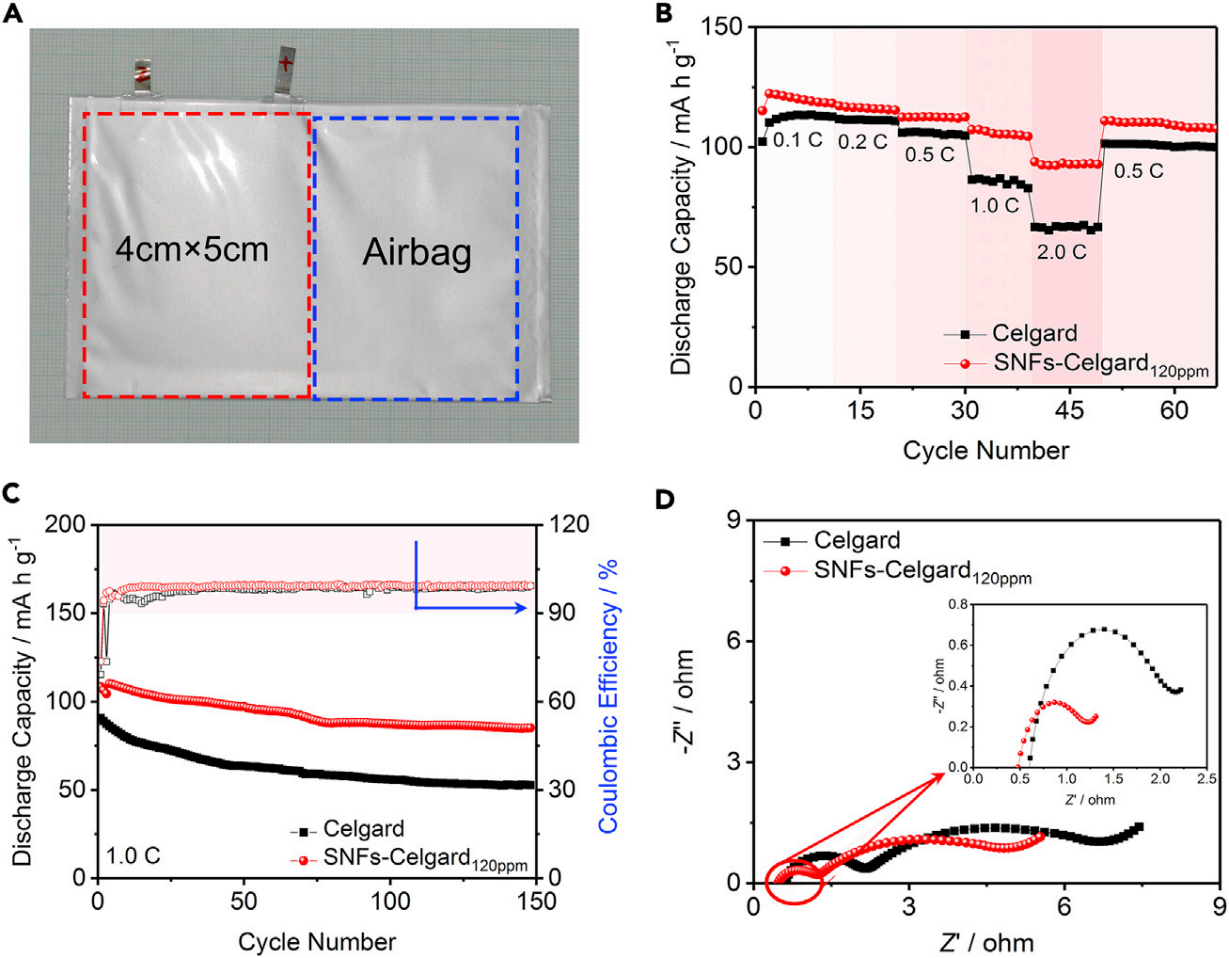
Electrochemical Performance of LiFePO_4_/Graphite Pouch Cells (A) Photograph of the pouch cell with the SNFs-Celgard_120ppm_ separator. (B–D) (B) Rate performance, (C) cycling stability at 1.0 C (1.0 C = 133 mA h g^−1^), and (D) Nyquist plots after 150 cycles.

To further assess usefulness of the SNFs-Celgard_120ppm_ separator in Li ion batteries, the LiFePO_4_/graphite pouch cells with a high areal electrode loading of 2.0 mA h cm^−2^ (15.4 mg cm^−2^) were tested (Figure 6). The capacity decay of the cell was 20.7% with increasing the rate from 0.1 to 2.0 C, whereas that of the cell with the Celgard separator reached 40.4%. The punch cell with the SNFs-Celgard_120ppm_ separator also showed much better cycling stability with a capacity decay of 21.4% at 1.0 C over 150 cycles and lower resistance.

In addition, the SNFs-Celgard separator has a chance of being acceptable to the battery industry, as the cost of the SNFs layer is only ∼0.11 USD m^−2^ (Table S5).

## Discussion

A superLEphilic/superhydrophobic and thermostable SNFs-Celgard separator was prepared by the growth of SNFs onto the Celgard separator. The superLEphilicity, LE uptake and retention rate, Li^+^ conductivity, superhydrophobicity, and moisture uptake of the separator are closely related to the microstructure of the SNFs, which can be controlled simply by the water concentration in toluene. The wettability of the separator is significantly different from all the reported separators. The separator has high superLEphilicity, fast LE diffusion, high LE uptake, LE retention rate, and Li^+^ conductivity. The separator also has high superhydrophobicity and low moisture uptake, making Li metal battery assembly simpler. Additionally, the separator has high thermostability. Consequently, the Li/LiFePO_4_ cells show high cycling stability, Coulombic efficiency, rate performance, and safety, as the separator could efficiently eliminate the side reactions at the interface of Li anode triggered by trace water and could reduce resistance of the cells. In addition, the separator outperforms the commercial ceramic-coated separators in the Li/LiFePO_4_ cells. Moreover, the separator could improve performance of the other Li metal batteries with high-voltage cathodes and the LiFePO_4_/graphite pouch cells. We believe that this work provides an avenue for designing advanced separators for Li batteries and other metal batteries. This study also opens up a new field of application of bioinspired superwetting surfaces, like oil/water separation did a decade ago.

### Limitations of the Study

The application of bioinspired superwetting surfaces on separators of Li metal batteries has not been well understood. Further in-depth study about the effects of superwetting separators on the performance of Li batteries should be carried out. In addition, the performance of the SNFs-Celgard separators was studied by using Li/LiFePO_4_ coin cells as opposed to pouch cells. Moreover, the O_2_-plasma activation technique may reduce the mechanical strength of polyolefin separators. Thus, new approaches for activation of polyolefin separators without sacrificing their inherent properties remain to be developed.

## Methods

All methods can be found in the accompanying Transparent Methods supplemental file.

## Supplemental Information

Document S1. Transparent Methods, Figures S1–S28, and Tables S1–S5
