# Supplementary material for: A SuperLEphilic/Superhydrophobic and Thermostable Separator Based on Silicone Nanofilaments for Li Metal Batteries
Source: iScience. 2019 Jun 11;16:420–32. doi: 10.1016/j.isci.2019.06.010 (PMC6593149; doi:10.1016/j.isci.2019.06.010)
Supplement: Document S1. Transparent Methods, Figures S1–S28, and Tables S1–S5 [file mmc1.pdf]

ISCI, Volume *16*

Supplemental Information

A SuperLEphilic/Superhydrophobic and Thermostable Separator Based on Silicone Nanofilaments for Li Metal Batteries

Yanfei Yang, Bucheng Li, Lingxiao Li, Stefan Seeger, and Junping Zhang

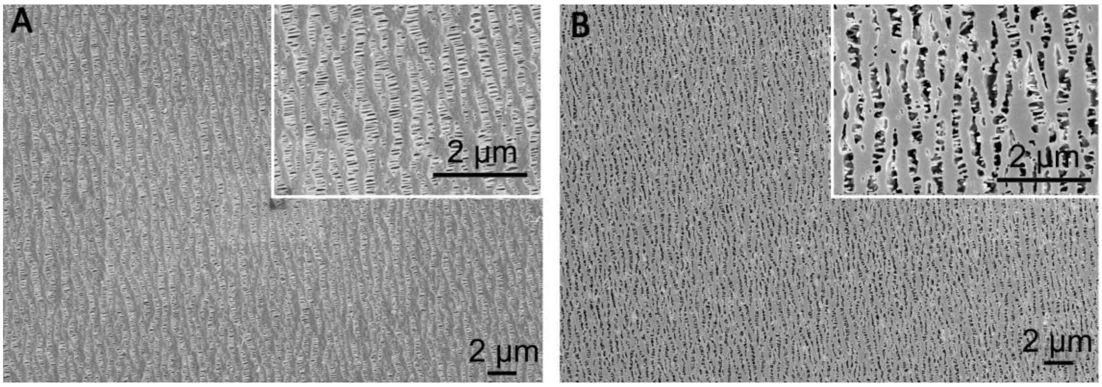

Figure S1. SEM images of the (A) Celgard and (B) O_2-plasma activated Celgard separators, related to Figure 1.

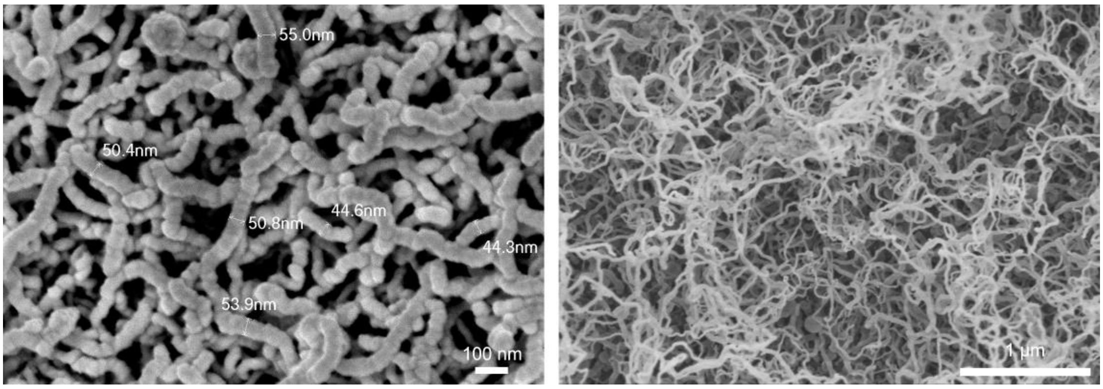

Figure S2. SEM images of the SNFs-Celgard$_{120ppm}$ separator, related to Figure 1B.

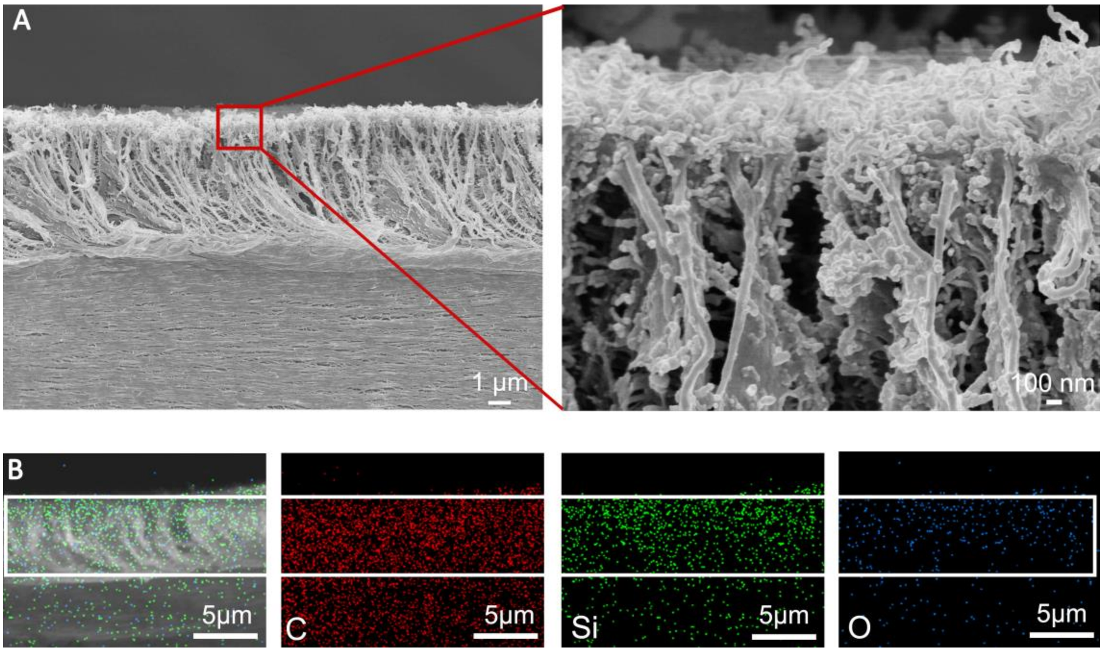

Figure S3. (A) Cross-sectional SEM images and (B) elemental maps of the SNFs-Celgard$_{120ppm}$ separator, related to Figure 1 and Figure 5.

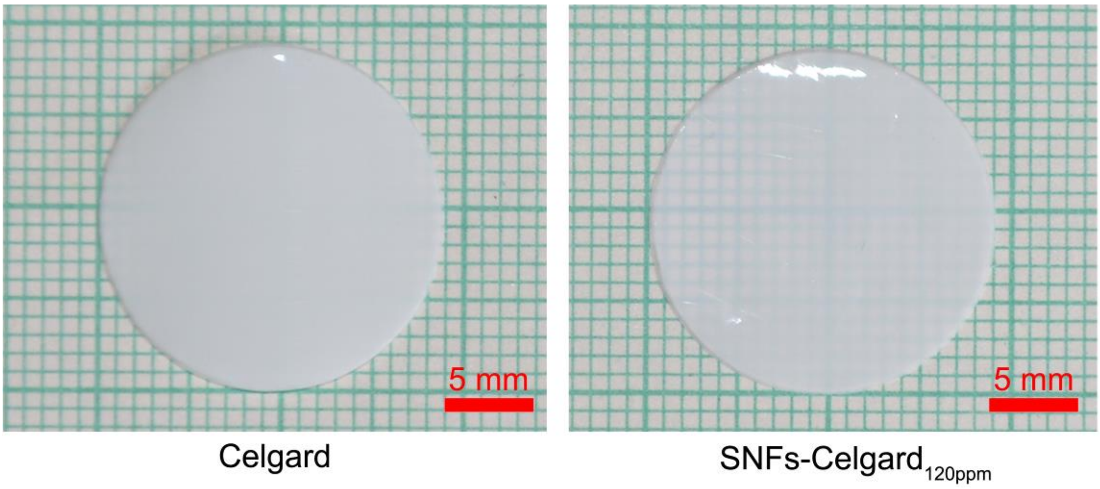

Figure S4. Photographs of the separators, related to Figure 1.

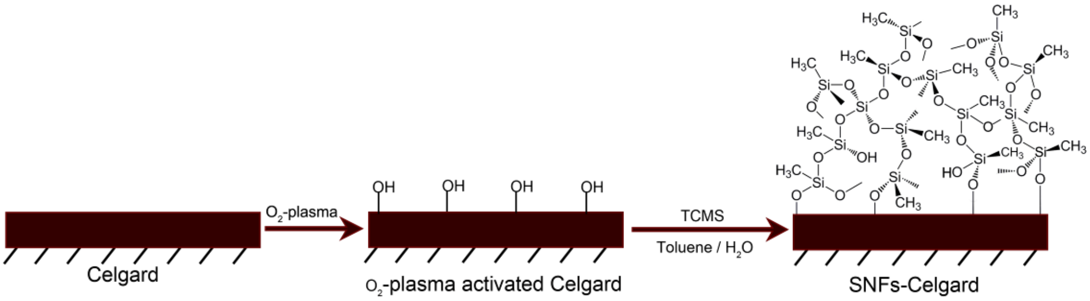

Figure S5. Synthesis of the SNFs-Celgard separator, related to Figure 1D-F.

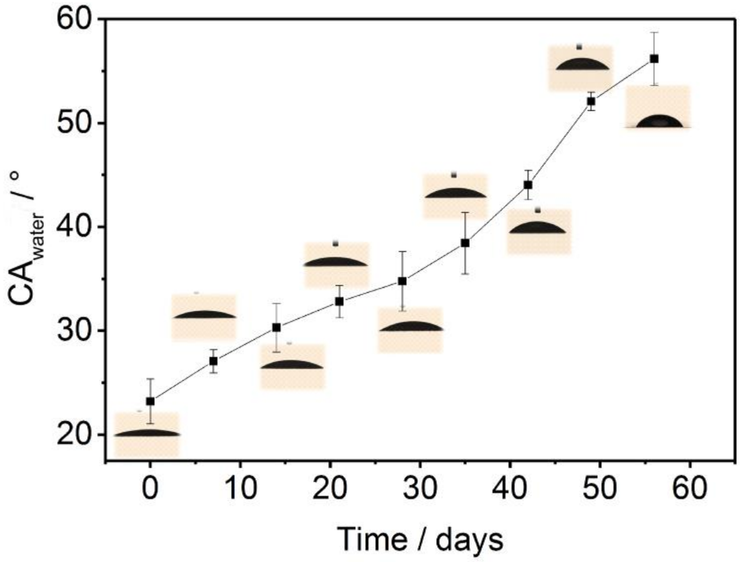

Figure S6. Variation of CA_{water} of the O_2-plasma activated Celgard separator with storage time (shown as means ± SD, n = 6), related to Figure 2F.

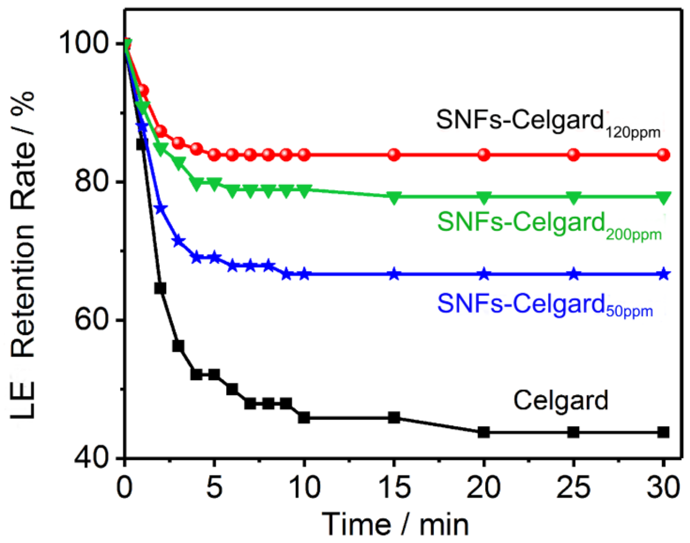

Figure S7. Variation of LE retention rate of the separators with storage time in room conditions, related to Figure 2K.

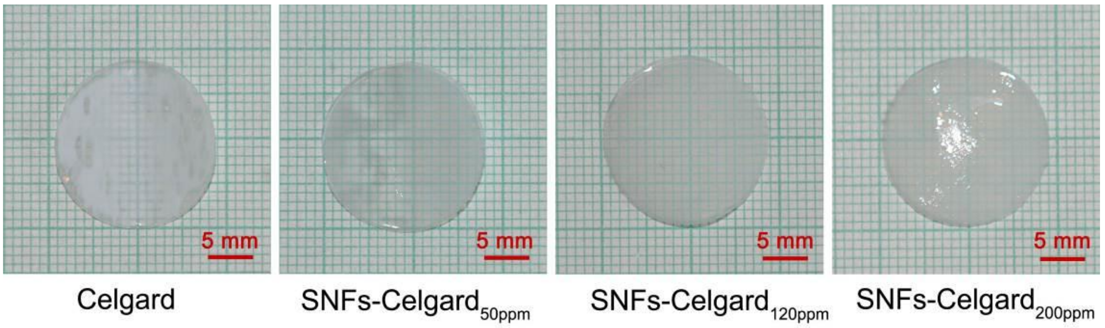

Figure S8. Photographs of the separators with absorbed LE after kept in room conditions for 1 h, related to Figure 2K.

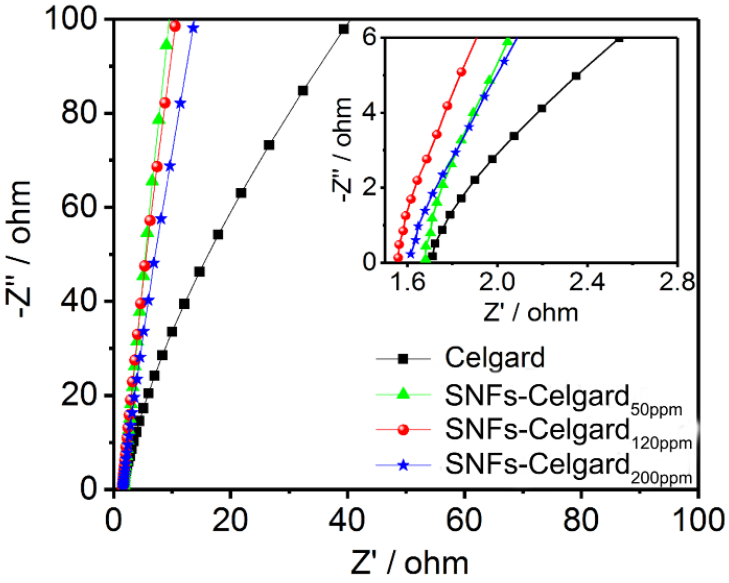

Figure S9. Impedance plots of the cells with different separators, related to Figure 2.

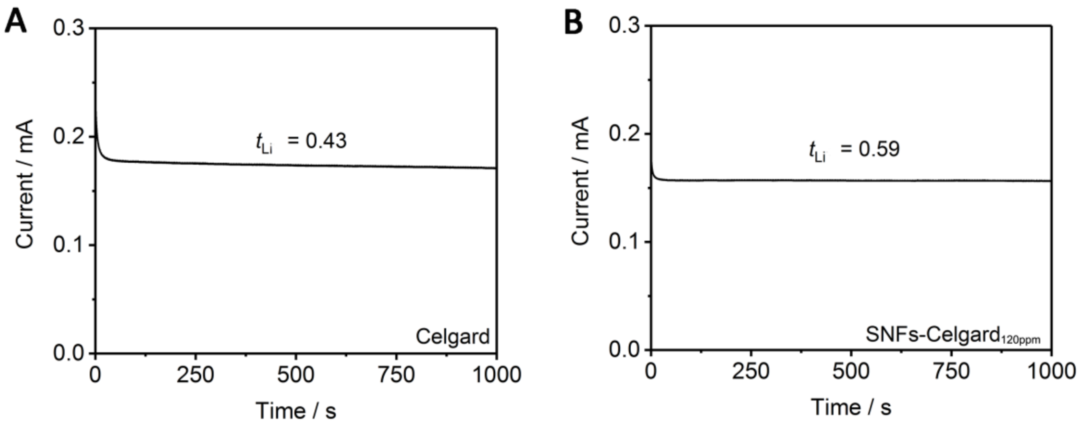

Figure S10. The characteristic of Li^+ transference number (t_{Li}), related to Figure 2.

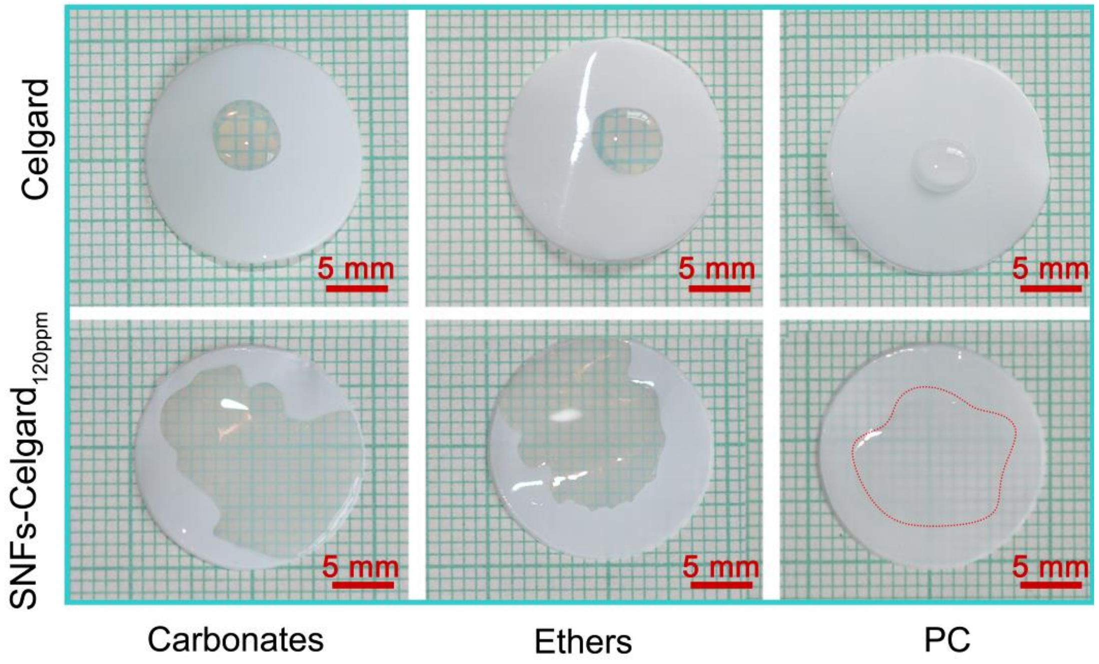

Figure S11. Photographs of wetting behavior of three commonly used LEs with different surface tensions on the surface of the Celgard and SNFs-Celgard$_{120ppm}$ separators, related to Figure 2P.

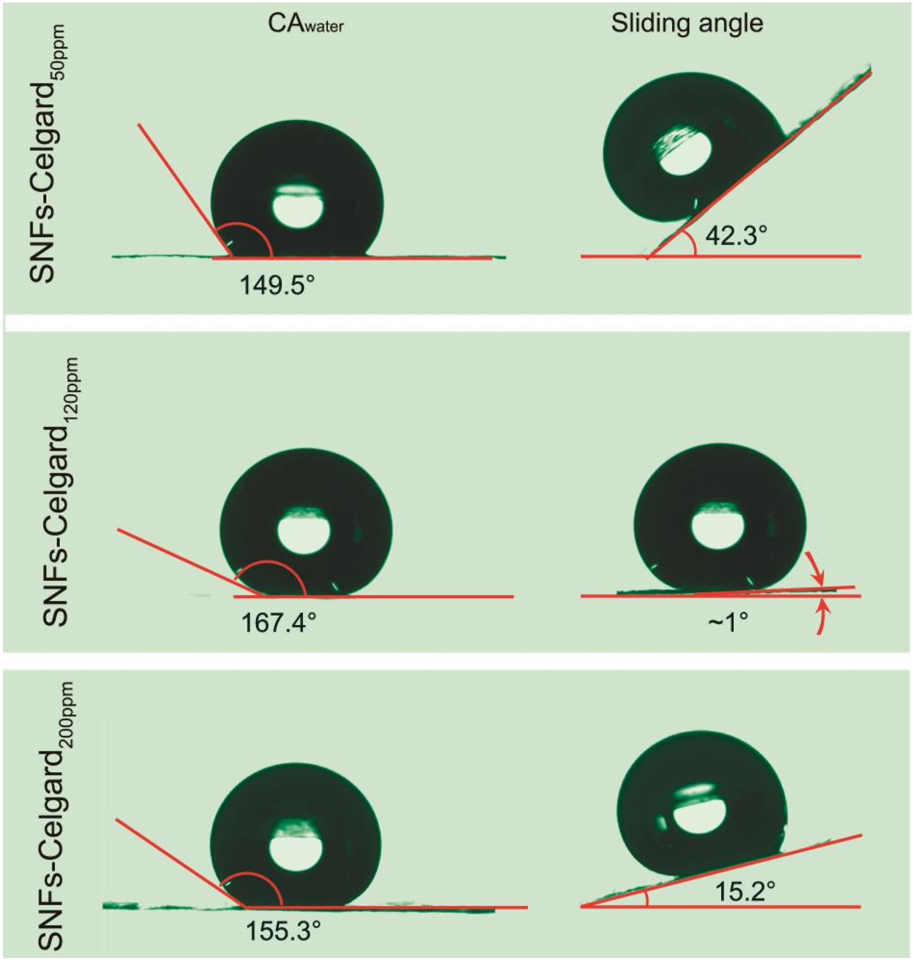

Figure S12. CA_{water} and sliding angle of water on the separators, related to Figure 3A.

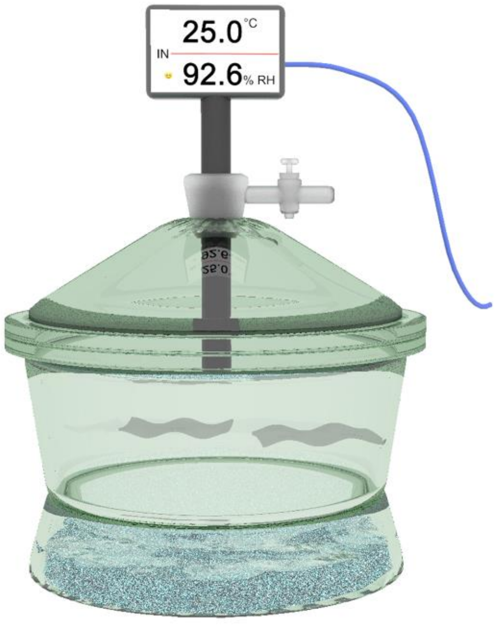

Figure S13. Schematic illustration of the device for measuring the moisture uptake of the separators, related to Figure 3C.

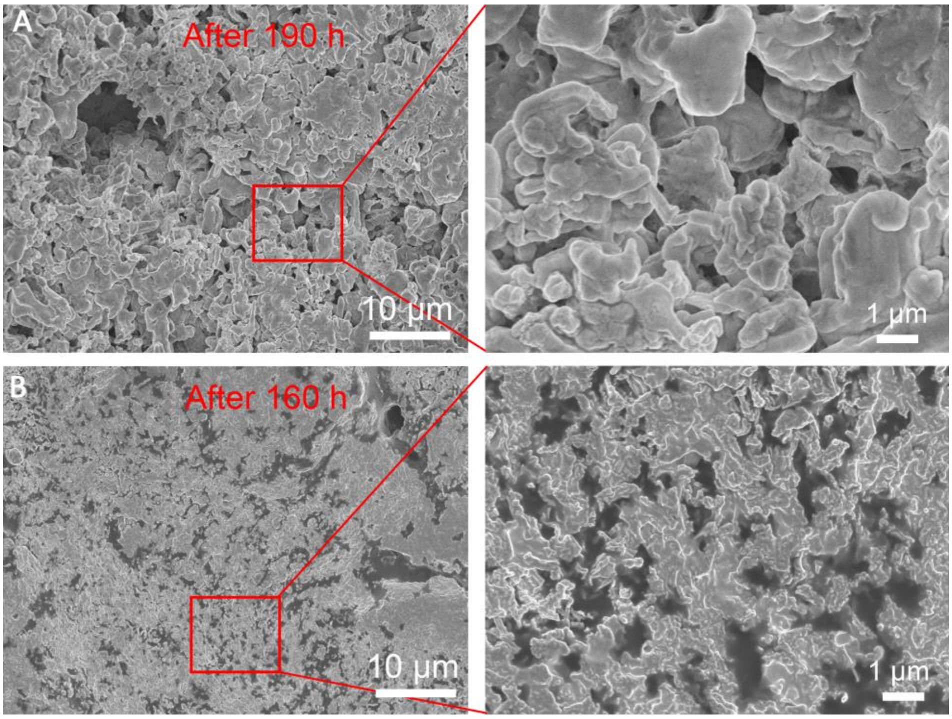

Figure S14. SEM images of the Li anode surface in the Li symmetric cells with (A) Celgard and (B) wet Celgard separators at a current density of 1.0 mA cm^{-2}, related to Figure 3D.

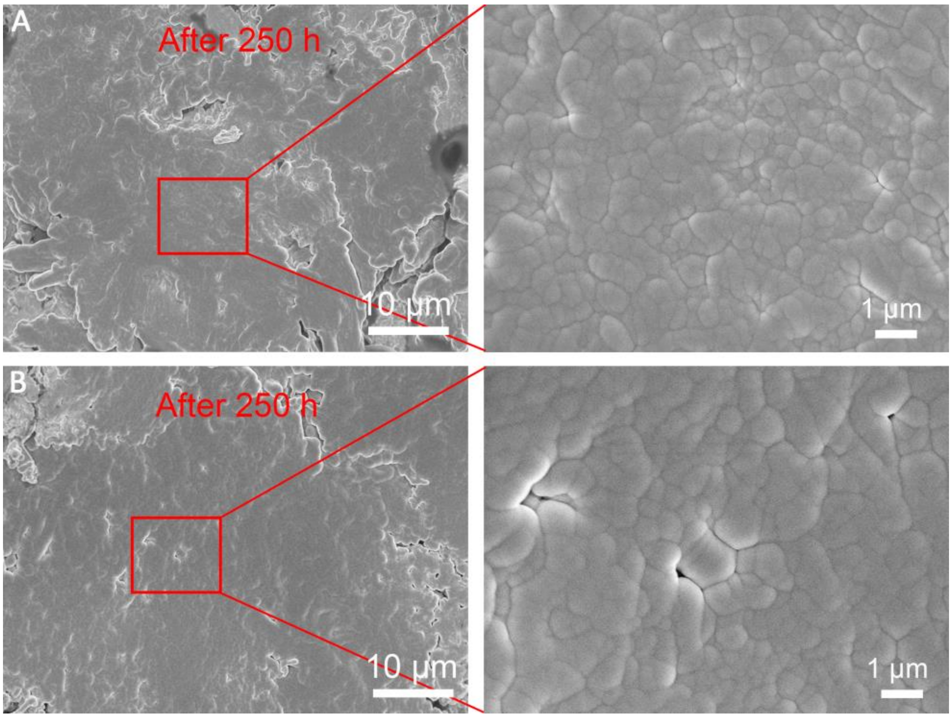

Figure S15. SEM images of the Li anode surface in the Li symmetric cells with (A) SNFs-$Celgard_{120ppm}$ and (B) wet SNFs-$Celgard_{120ppm}$ separators at a current density of 1.0 mA cm^{-2}, related to Figure 3F.

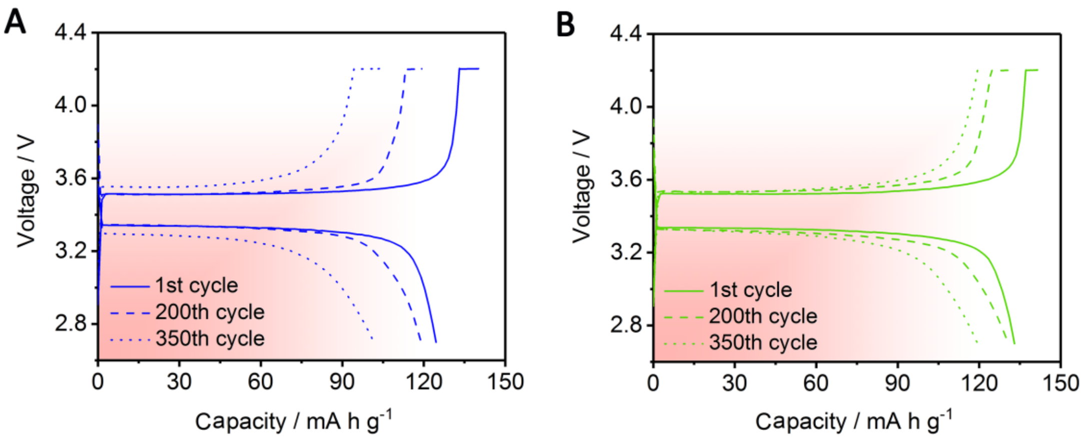

Figure S16. Charge/discharge curves of the Li/$LiFePO_4$ cells with (A) SNFs-$Celgard_{50ppm}$ and (B) SNFs-$Celgard_{200ppm}$ separators, related to Figure 4A.

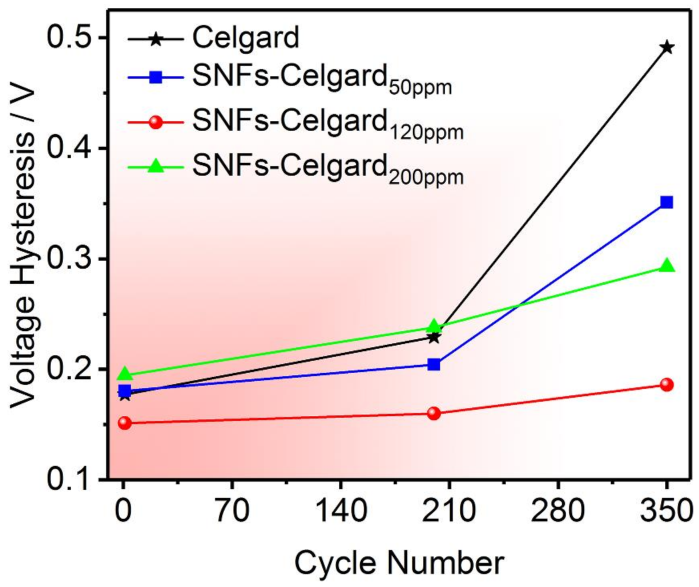

Figure S17. Voltage hysteresis of the Li/$LiFePO_4$ cells with cycle number, related to Figure 4B-C.

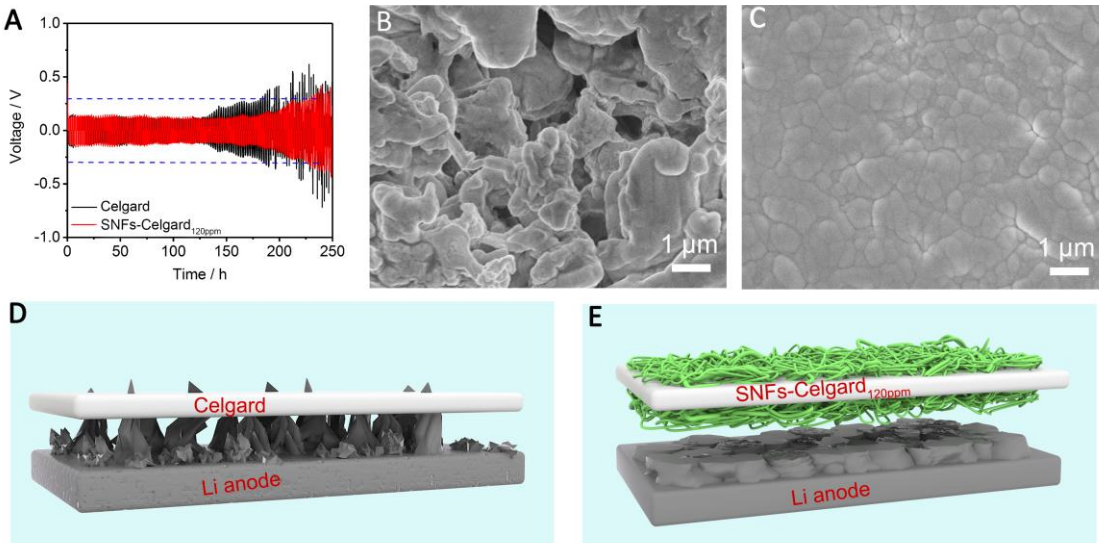

Figure S18. Electrochemical performances of the Li symmetric cells with different separators, related to Figure 4A. (A) Voltage-time curves. SEM images of the Li anode surface in the Li symmetric cells with (B) the Celgard and (C) SNFs-Celgard$_{120ppm}$ separator. The amount of plated Li is 1.0 mAh cm^{-2}, and the current density is 1.0 mA cm^{-2} in each cycle. (D, E) Schematic illustrations of dendrite Li growth with different separators.

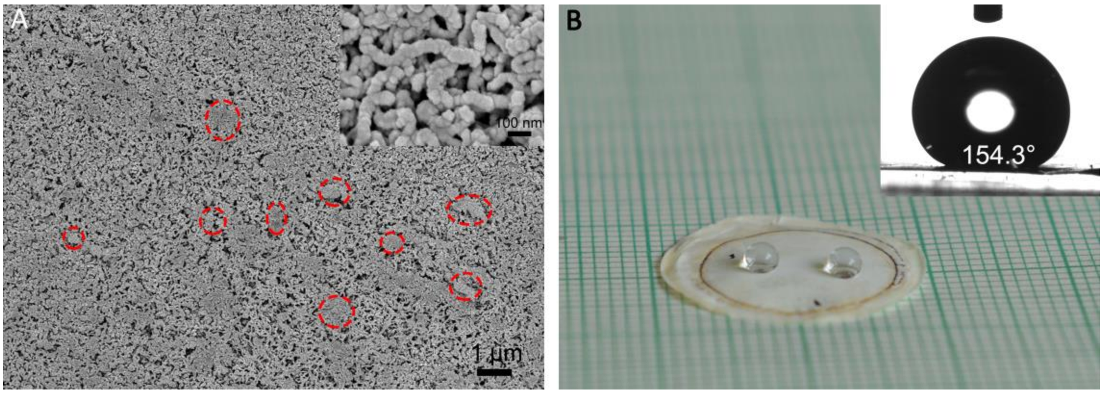

Figure S19. (A) SEM images of the SNFs-Celgard$_{120ppm}$ separator after 350 cycles and (B) photographs of 10 μL water droplets on the SNFs-Celgard$_{120ppm}$ separator after 350 cycles, related to Figure 4.

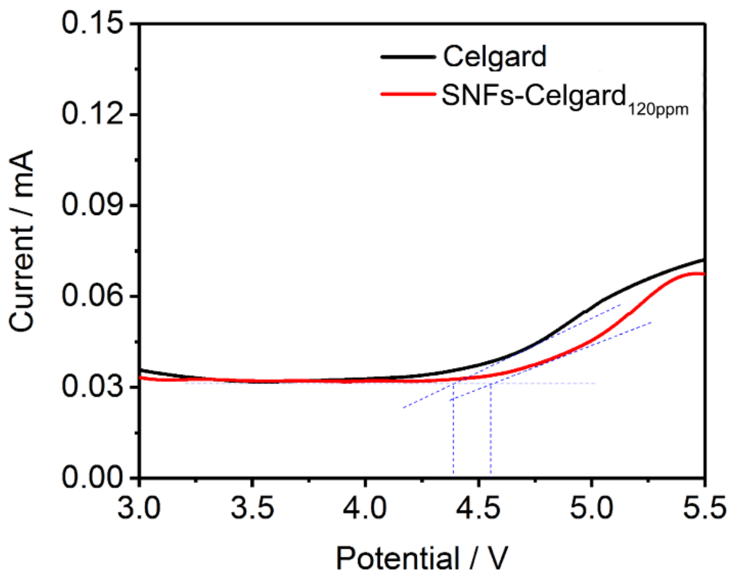

Figure S20. Linear sweep voltammetry profiles of the Celgard and SNFs-Celgard$_{120ppm}$ separators, related to Figure 4.

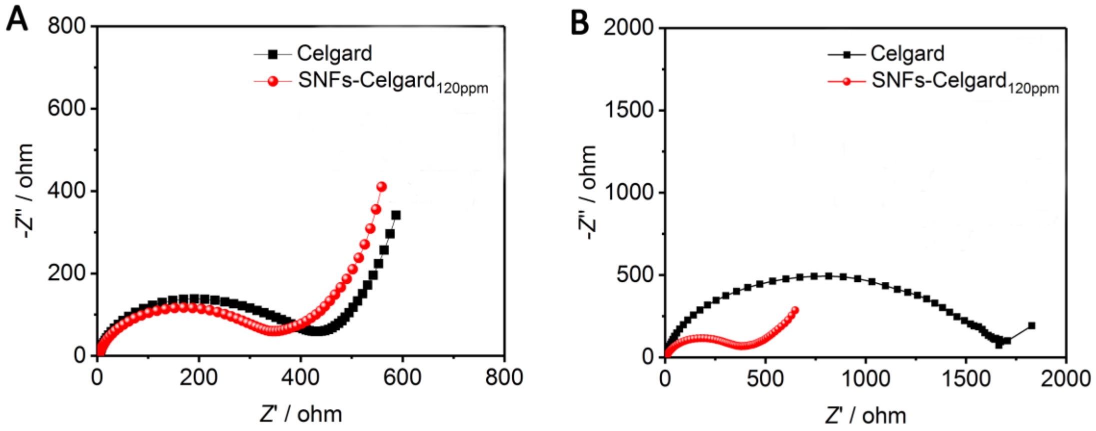

Figure S21. Nyquist plots of the Li/$LiFePO_4$ cells with different separators (A) before cycling and (B) after 100 cycles, related to Figure 4A.

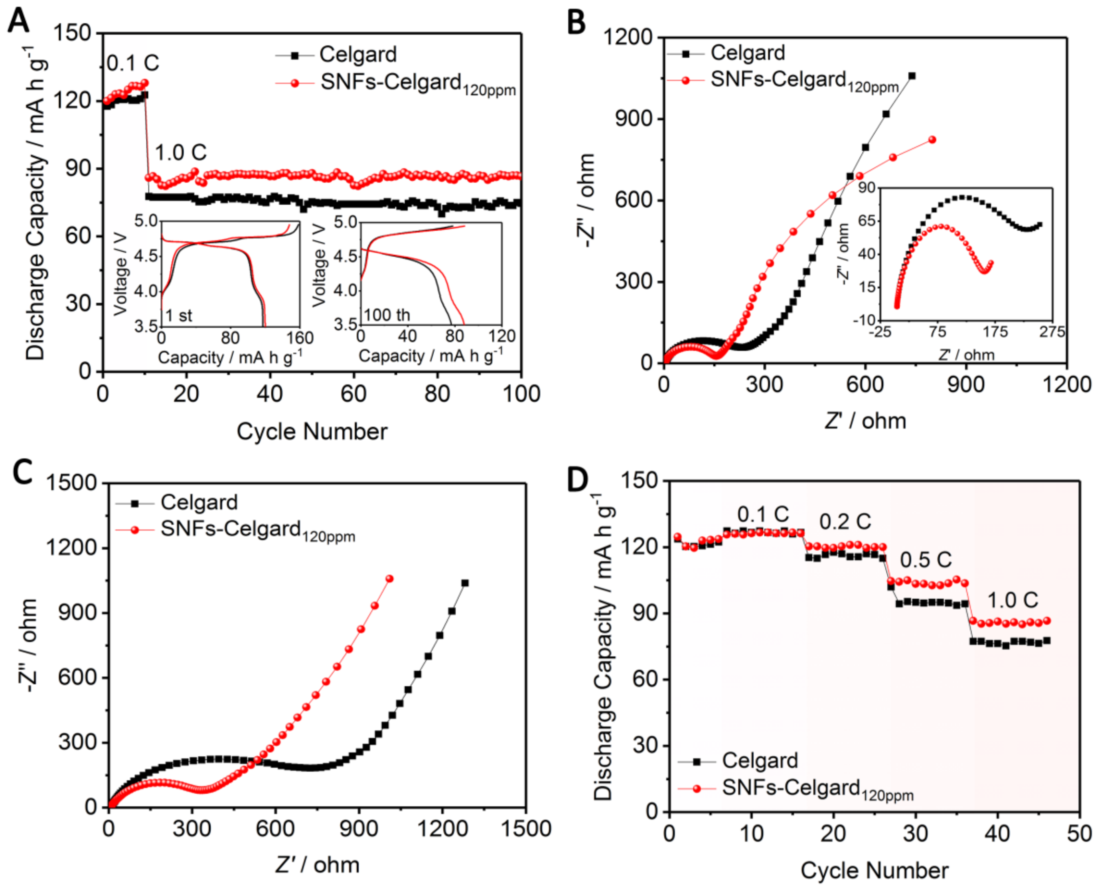

Figure S22. Electrochemical performances of the Li/$LiNi_{0.5}Mn_{1.5}O_4$ cells, related to Figure 4. (A) Cycling stability at 1.0 C (1.0 C = 140 mA h g^{-1}), Nyquist plots (B) before cycling and (C) after 100 cycles, and (D) rate performance. The insets in (A) show discharge/charge profiles.

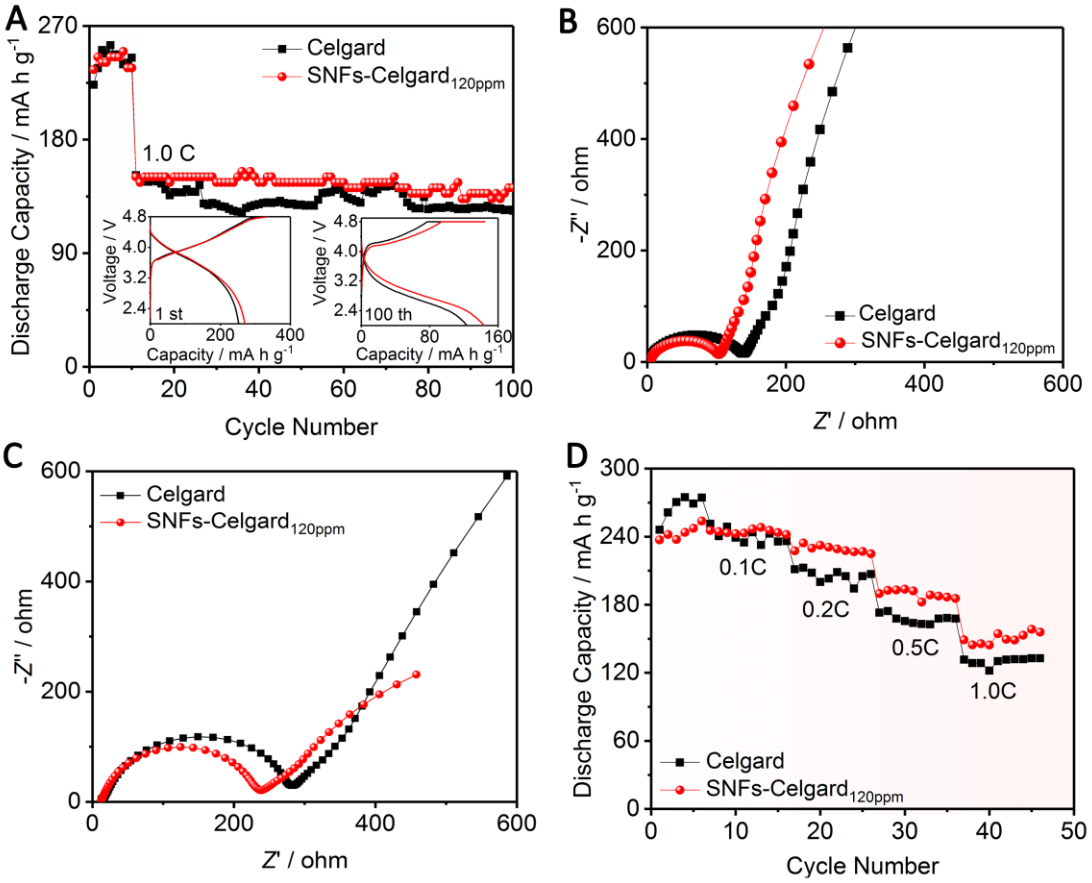

Figure S23. Electrochemical performances of the Li/$Li_{1.2}Mn_{0.54}Ni_{0.3}Co_{0.3}O_2$ cells, related to Figure 4. (A) Cycling stability at 1.0 C (1.0 C = 360 mA h g^{-1}), Nyquist plots (B) before cycling and (C) after 100 cycles, and (D) rate performance. The insets in (A) show discharge/charge profiles.

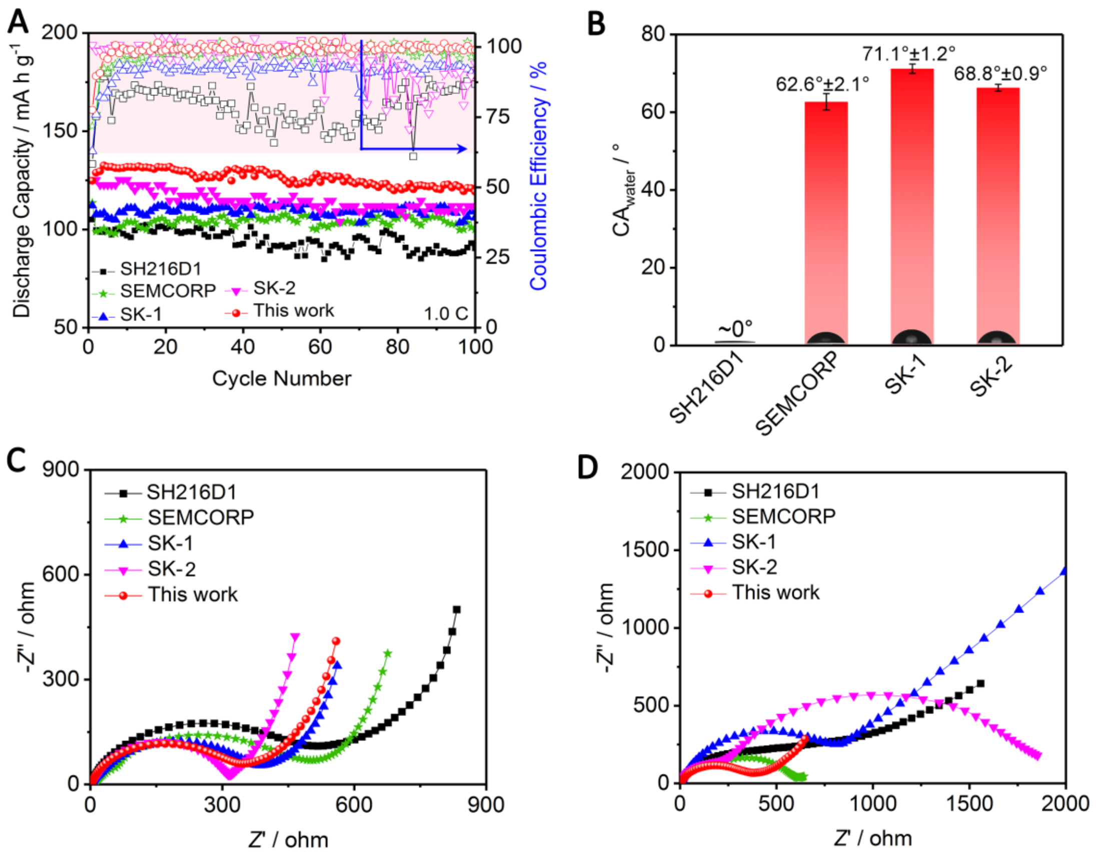

Figure S24. Electrochemical Performances of the Li/$LiFePO_4$ cells with the SNFs-Celgard$_{120ppm}$ and the ceramic coated separators, related to Figure 4. (A) Cycling stability at 1.0 C (1.0 C = 160 mA h g^{-1}), (B) CA_{water} on the ceramic coated separators (shown as means ± SD, n = 6), Nyquist plots of the cells (C) before cycling and (D) after 100 cycles. We chose four commercial ceramic coated separators: (1) SEMCORP (one side Al_2O_3 coated PE membrane with a coating thickness of 4 ± 0.1 µm, Yunnan Energy New Material Co., LTD, China), (2) SH216D1 (one side Al_2O_3 coated PP membrane with a coating thickness of 4±1 µm, Shenzhen Senior Technology Material Co., LTD, China), (3) SK-1 (one side Al_2O_3 coated PE membrane with a coating thickness of 4 ± 0.1 µm SKinnovation, Korea) and (4) SK-2 (double side Al_2O_3 coated PE membrane with a coating thickness of 2 µm on each side, SKinnovation, Korea).

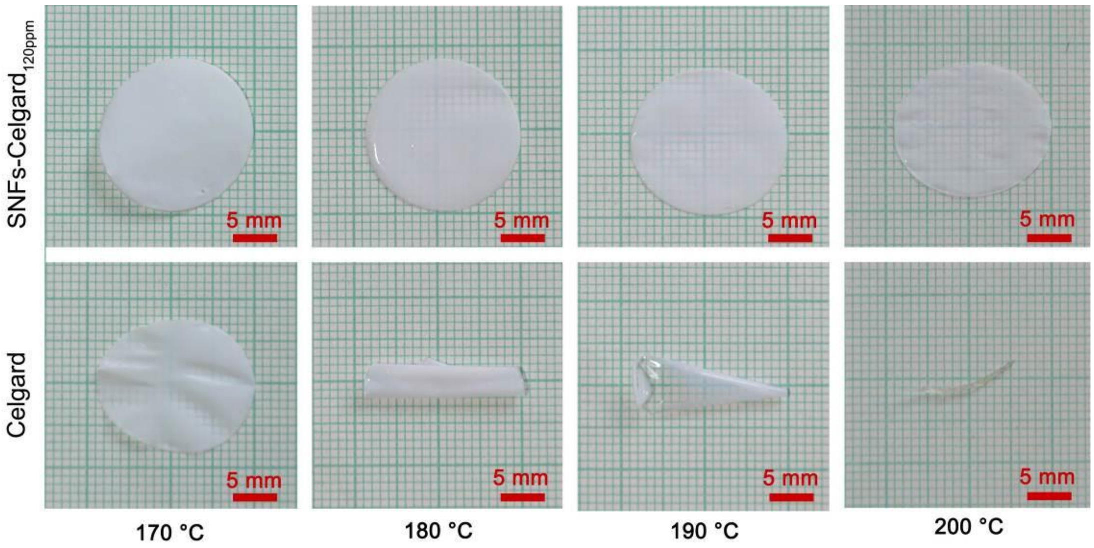

Figure S25. Dimension of the separators before and after heat treatment at different temperature for 1 h, related to Figure 5A.

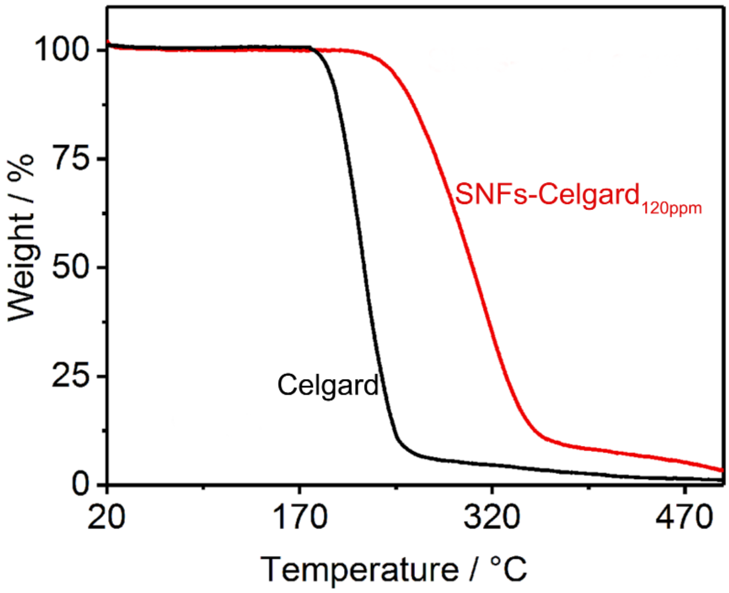

Figure S26. TGA curves of the separators, related to Figure 5.

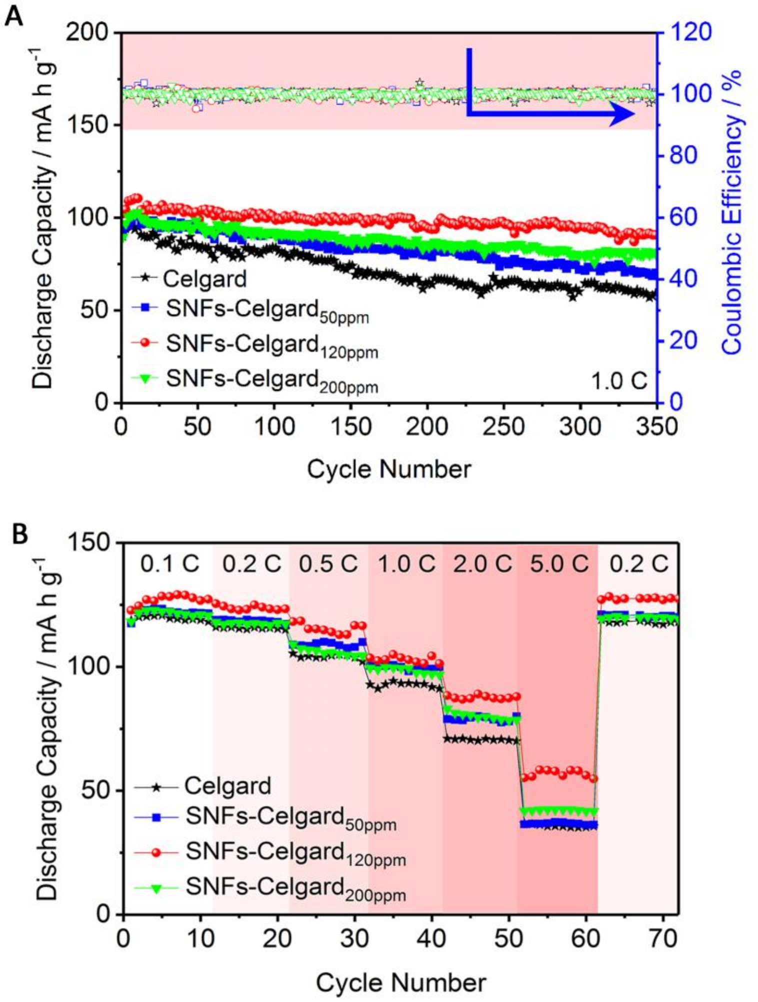

Figure S27. (A) Cycling stability and (B) rate performance of the $LiFePO_4$/graphite cells with different separators (1.0 C = 133 mA h g^{-1}), related to Figure 6.

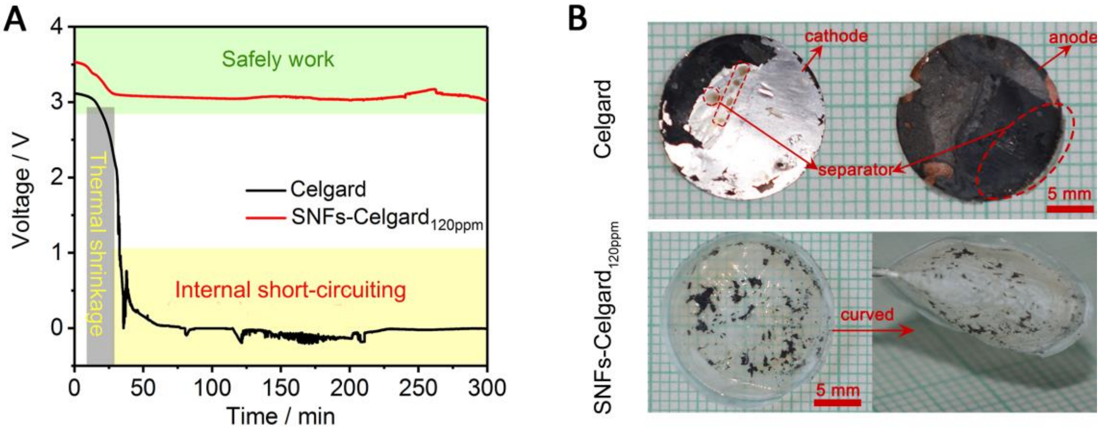

Figure S28. (A) OCV curves of the $LiFePO_4$/graphite cells with different separators at 170 °C. (B) Photographs of the separators after the OCV test, related to Figure 6.

Table S1. Surface tension of various LEs (shown as means ± SD, n = 12), related to Figure 2 and Figure 3.

LEs	Carbonates	Ethers	PC
Surface tension / mN m^{-1}	27.79 ± 0.06	26.56 ± 0.35	31.35 ± 0.16

Table S2. Atomic ratio of O, C and Si on the surface of the SNFs-$Celgard_{120ppm}$ separator, related to Figure 1E and F.

Elements	O	C	Si
Atomic ratio / %	31.55	46.78	21.67

Table S3. Physical and electrochemical parameters of the separators, related to Figure 2.

Separators	Celgard	SNFs-$Celgard_{50ppm}$	SNFs-$Celgard_{120ppm}$	SNFs-$Celgard_{200ppm}$
Porosity / %	44.9	45.7	51.9	47.9
LE uptake / %	91.3	165.5	287.8	196.9
Conductivity / mS cm^{-1}	0.727	0.740	1.02	0.832

Table S4. Wettability of the previously reported separators and the SNFs-Celgard$_{120ppm}$ separator, related to Figure 2 and Figure 3.

Separators	CA_{LE} / °	CA_{water} / °	Ref.
PVDF/ZSM-Si(Al) coated PE	- [a]	14.5	Mao et al., 2017
(PEI/SiO_2) modified PE	-	24.6	Wang et al., 2015
PE-SiO_2@PDA	-	36	Dai et al., 2016
$(PAA/ZrO_2)_3$ modified PE	-	38	Xu et al., 2015
Polydopamine coated PE	-	39	Ryou et al., 2011
N-SiO_2 coated PE	-	51.3	Cho et al., 2017
PAA/PEO@PP	27.8	71.7	Li et al., 2015
Tannic acid coated PP	-	72	Pan et al., 2015
SiO_2 grafted PE	-	79	Zhu et al., 2015
TiO_2 grafted PE	-	89	Zhu et al., 2016
Al_2O_3/LPMA64 coated PE	-	95	Na et al., 2016
Al_2O_3/PI coated PE	0	Hydrophilic [b]	Shi et al., 2016
SiO_2-PMMA coated PE	6.1	hydrophilic	Yang et al., 2015
SiO_2/WCDA coated PE	8	hydrophilic	Chen et al., 2016
Al_2O_3/CGS grafted PE	21	hydrophilic	Jiang et al., 2017
Hydroxyapatite/cellulose fibers	0	hydrophilic	Li et al., 2017
PU	5	hydrophilic	Kim et al., 2016
Chitin nanofibers	9.2	hydrophilic	Zhang et al., 2017
Poly(*p*-phenylene benzobisoxazole)	20	hydrophilic	Hao et al., 2016
PPy@NCFs/NCFs	0	hydrophilic	Wang et al., 2017
SH216D1	4	0	tested [c]
SEMCORP	0	62.6	tested
SK-1	0	71.1	tested
SK-2	8.3	68.8	tested
**SNFs-Celgard$_{120ppm}$**	**0**	**167.4**	**This work**

a. "-" means not mentioned.

b. "hydrophilic" means that the material for modification of the separators is hydrophilic.

c. "tested" means that the CA of LE and water was measured at 25 °C using 10 μL droplets on a Contact Angle System OCA 20.

Table S5. Cost of the SNFs layer on the SNFs-Celgard separator, related to Figure 1.

Cost of TCMS	Cost of O_2-plasma treatment	Cost of the SNFs layer on the SNFs-Celgard separator
152.5 USD for 2 kg (ABCR)	0.03 USD m^{-2}	0.58 USD m^{-2}
144.0 USD for 2 kg (Gelest)		0.55 USD m^{-2}
10430 USD for 1000 kg (block transaction)		0.11 USD m^{-2}

TRANSPARENT METHODS

Materials

Toluene, ethanol and *n*-hexadecane were purchased from China National Medicines Co., Ltd. TCMS (98%) was purchased from Gelest. $LiFePO_4$, carbon black, graphite, *N*-methyl-2-pyrolidone and PVDF were purchased from Shenzhen Kejing Star Technology Co., Ltd., China. All the LEs were purchased from Zhangjiagang Guotai Huarong Chemical New Material Co., Ltd., China. $LiNi_{0.5}Mn_{1.5}O_4$ and $Li_{1.2}Mn_{0.54}Ni_{0.3}Co_{0.3}O_2$ were provided by Lanzhou University of Technology, China. All chemicals were used as received without further purification.

Preparation of SNFs-Celgard Separators

First, the polyolefin separator (Celgard 2400) was activated with O_2-plasma (HARRICK PLASMA PDC-002, USA) at an O_2 flow rate of 60 sccm and a power of 15 W for 1 min. The SNFs-Celgard separators were prepared using a similar method described in our previous papers (Zhang and Seeger, 2011; Zhang et al., 2014). In brief, a fresh solution containing 80 mL of toluene with certain water concentrations (50, 120, and 200 ppm) and 50 µL of TCMS was prepared in a media bottle. Subsequently, a piece of the O_2-plasma activated Celgard separator (68.75 cm^2) was immersed in the solution at room temperature. 6 h later, the SNFs-Celgard separator was successfully prepared. The separator was successively rinsed with 10.0 mL of toluene, 10.0 mL of ethanol and 10 mL of water/ethanol mixture (1:1, v/v), and then dried at 30 °C.

Measurements of Wetting Behaviors

To investigate wettability of the separators, the CA of water and LEs was collected at 25 °C using 10 µL droplets on a Contact Angle System OCA 20 (Dataphysics, Germany). It should be noted that the LE refers to the carbonates based LE (1 M $LiPF_6$ in 1:1 (v/v) EC and DMC) if not specified. The surface tension of various LEs was measured at 25 °C using 10 µL droplets on a Contact Angle System DSA100 (KRÜSS,

Germany). The dynamic wetting behavior of the separators was tested at 4000 fps using a high-speed digital camera (FASTCAM Mini UX100, Photron, Japan).

Measurements of LE Uptake and Moisture Uptake

The LE uptake of the separators was measured by soaking the separators in the LE for 2 h. Subsequently, the separators were taken out of the LE, and the excess LE on the surface of the separator was removed by wiping with filter paper. The LE uptake was calculated using formula (Dai et al., 2016):

$$\text{LE uptake (\%)} = (m_2\text{-}m_1)/m_1 \times 100\% \quad (1)$$

where m_1 and m_2 are the weight of the separators before and after LE absorption, respectively.

To measure the moisture uptake of the separators, the separators were dried at 60 °C for 24 h in a vacuum oven to a constant weight. Subsequently, the separators were put into a chamber with a constant relative humidity of 92.6% at 25 °C for 7 days (Figure S13). The moisture uptake was calculated using formula (Mihranyan et al., 2004; Peng et al., 2013):

$$\text{Moisture uptake (\%)} = (m_4\text{-}m_3)/m_3 \times 100\% \quad (2)$$

where m_3 is the weight of the dry separators, and m_4 is the weight of conditioned separators (termed as wet ones). In order to study the effect of moisture uptake on performance of the separators, the separators after moisture uptake test were immediately used for assembly of Li symmetric cells.

Electrode Preparation

To preparation the $LiFePO_4$ cathode, the $LiFePO_4$ powder was mixed with 10 wt% PVDF and 10 wt% super P in *N*-methyl-2-pyrolidone to form the active material slurry. The slurry was coated on Al foil by the tape casting method. The cathode was dried in a vacuum oven at 60 °C for 24 h, compressed, and cut into circular disks. The content of the active material in each cathode circular disk is 2.6 mg cm^{-2}. The high voltage $LiNi_{0.5}Mn_{1.5}O_4$ and $Li_{1.2}Mn_{0.54}Ni_{0.3}Co_{0.3}O_2$ cathodes were prepared via a similar process. The $LiNi_{0.5}Mn_{1.5}O_4$ and $Li_{1.2}Mn_{0.54}Ni_{0.3}Co_{0.3}O_2$ contents in the

cathodes are 2.0 mg cm^{-2}. The graphite anode was prepared via a similar process, except that the carboxymethylcellulose was used as a binder and the slurry was coated on copper foil. The content of graphite in each anode circular disk is 4.5 mg cm^{-2}.

Cell Assembly and Electrochemical Tests

CR2032 coin cells were used for all cell assembly and electrochemical tests. 50 μL of the LE was used in each cell. All the procedures were carried out in an Ar-filled glovebox with O_2 and water contents below 0.1 ppm. The Li symmetrical cells were assembled by sandwiching a separator between two Li electrodes. The performance of the Li symmetric cells was recorded using a CT2001A battery test system (LAND Electronic Co., Ltd. China) at 1 mA cm^{-2} and the amount of plated Li is 1.0 mA h cm^{-2}. For the Li metal cells, the $LiFePO_4$, $LiNi_{0.5}Mn_{1.5}O_4$ and $Li_{1.2}Mn_{0.54}Ni_{0.3}Co_{0.3}O_2$ cathodes, and the separators were dried at 60 °C for 2 h in a vacuum oven prior to cell assembly, and the cells were assembled using different cathodes and Li anode with the separators. The $LiFePO_4$/graphite cells were assembled using $LiFePO_4$ cathode and graphite anode with the separators. The pouch cells were assembled using $LiFePO_4$ cathode and graphite anode. The areal electrode loading and size of the cathode were 15.4 mg cm^{-2} (2.0 mA h cm^{-2}) and 20 cm^2 (4 cm × 5 cm), respectively. The assembled cells were aged at room temperature for 24 h before testing. The discharge/charge and cycling data were recorded using a CT2001A battery test system with a certain voltage window at various cycling rate. The voltage window is 2.7-4.2 V for the Li/$LiFePO_4$ cells, 3.0-4.9 V for the Li/$LiNi_{0.5}Mn_{1.5}O_4$ cells, 2.4-4.8 V for the Li/$Li_{1.2}Mn_{0.54}Ni_{0.3}Co_{0.3}O_2$ cells and 2.4-3.7 V for the $LiFePO_4$/graphite cells.

The electrochemical stability of the separators was evaluated using linear sweep voltammetry (CHI660E, Chenhua Instruments Co., China) from 3.0 to 5.0 V (vs. Li/Li^+) at a scan of 1 mV s^{-1}. The cell was assembled by sandwiching a separator between stainless steel (working electrode) and Li metal (reference and counter electrodes). The Li^+ conductivity of the separators was calculated by the electrochemical impedance spectroscopy (EIS). The cells were assembled by sandwiching a separator

between two stainless steel blocking electrodes. The EIS measurements were carried out using an impedance analyzer (CHI660E) at open circuit potential with a constant perturbation amplitude of 5 mV in the frequency range of 0.1-100 KHz, and was calculated using formula (Yang and Zhang, 2018):

$$\sigma = L/(R_b \times A_1) \qquad (3)$$

where σ is the Li^+ conductivity (mS cm^{-1}), L is the thickness of the separator (cm), R_b is the bulk resistance (Ω), and A_1 is the area of the stainless steel electrode (cm^2).

The Li^+ transference number (t_{Li}) of the separators was measured by a potentiostatic polarization method with a constant potential at 20 mV for 1000 s, and was calculated using formula (He et al., 2018):

$$t_{Li} = I_s\ (\Delta V - I_o R_o)/I_o(\Delta V - I_s R_s) \qquad (4)$$

where ΔV is the potentiostatic potential (V); I_o and I_s are the current at initial and steady state (mA), respectively; R_o and R_s are the resistance before and after the potentiostatic polarization (Ω), respectively.

In order to study the safety performance of the cells with different separators at high temperature, the open circuit voltage (OCV) measurement was carried out. The cells were assembled using $LiFePO_4$ cathode and Li anode or graphite anode with different separators. The cell was charged to 4.0 V at 25 °C, and then put into an oven at certain temperature. Subsequently, variation of the voltage with time was monitored using an electrochemical working station (CHI660E).

Characterization

Surface morphology of the separators was observed via field emission scanning electron microscopy (SEM, JSM-6701F, JEOL). Fourier transform infrared (FTIR) spectra of the separators were collected using a Nicolet NEXUS FTIR spectrometer using KBr pellets. X-ray photoelectron spectra (XPS) of the separators were recorded using a VG ESCALAB 250 Xi spectrometer with a monochromated Al K_α X-ray radiation source and a hemispherical electron analyzer. The spectra were collected in the constant pass energy mode with a value of 100 eV, and all binding energies were calibrated using the C 1s peak at 284.6 eV as the reference. The porosity of the

separators was measured by soaking the separators in *n*-hexadecane for 2 h, and supposing that the pore volume of the separator is equal to the volume of the absorbed *n*-hexadecane by the separator (Dai et al., 2016). The excess *n*-hexadecane on the surface of the separator was removed by wiping with filter paper. The porosity was calculated using formula 5:

$$\text{Porosity (\%)} = (\Delta m/\rho)/V_o \quad (5)$$

where Δm is the mass difference of the separator before and after immersion in *n*-hexadecane, V_o is the total volume of the separator, and ρ is the density of *n*-hexadecane.

In order to study thermostability of the separators, the thermal shrinkage was measured based on the area change of the separator (2.83 cm^2 circle) before and after heat treatment at certain temperature (120-200 °C) for 1 h, and was calculated using formula 6:

$$\text{Thermal shrinkage (\%)} = (A_1\text{-}A_2)/A_1 \times 100\% \quad (6)$$

where A_1 and A_2 are the areas of the separator before and after heat treatment, respectively. The thermostability was also analyzed by differential scanning calorimetry (DSC) and thermal gravimetric analysis (TGA) at a heating rate of 10 °C min^{-1} from 20 to 500 °C in O_2 atmosphere.

SUPPLEMENTAL REFERENCES

Chen, W., Shi, L., Zhou, H., Zhu, J., Wang, Z., Mao, X., Chi, M., Sun, L., and Yuan, S. (2016). Water-based organic-inorganic hybrid coating for a high-performance separator. ACS Sus. Chem. Eng. *4*, 3794-3802.

Cho, J., Jung, Y.C., Lee, Y.S., and Kim, D.W. (2017). High performance separator coated with amino-functionalized SiO_2 particles for safety enhanced lithium-ion batteries. J. Membr. Sci. *535*, 151-157.

Dai, J., Shi, C., Li, C., Shen, X., Peng, L., Wu, D., Sun, D., Zhang, P., and Zhao, J. (2016). A rational design of separator with substantially enhanced thermal features for lithium-ion batteries by the polydopamine-ceramic composite modification of polyolefin membranes. Energy Environ. Sci. *9*, 3252-3261.

Hao, X., Zhu, J., Jiang, X., Wu, H., Qiao, J., Sun, W., Wang, Z., and Sun, K. (2016). Ultrastrong polyoxyzole nanofiber membranes for dendrite-proof and heat-resistant battery separators. Nano Lett. *16*, 2981-2987.

He, Y., Chang, Z., Wu, S., Qiao, Y., Bai, S., Jiang, K., He, P., and Zhou, H. (2018). Simultaneously inhibiting lithium dendrites growth and polysulfides shuttle by a flexible MOF-based membrane in Li-S batteries. Adv. Energy Mater. *8*, 1802130.

Jiang, X., Zhu, X., Ai, X., Yang, H., and Cao, Y. (2017). Novel ceramic-grafted separator with highly thermal stability for safe lithium-ion batteries. ACS Appl. Mater. Interfaces *9*, 25970-25975.

Kim, B.G., Kim, J.S., Min, J., Lee, Y.H., Choi, J.H., Jang, M.C., Freunberger, S.A., and Choi, J.W. (2016). A moisture- and oxygen-impermeable separator for aprotic Li-O_2 batteries. Adv. Funct. Mater. *26*, 1747-1756.

Li, B., Li, Y., Dai, D., Chang, K., Tang, H., Chang, Z., Wang, C., Yuan, X.Z., and Wang, H. (2015). Facile and nonradiation pretreated membrane as a high conductive separator for Li-Ion batteries. ACS Appl. Mater. Interfaces *7*, 20184-20189.

Li, H., Wu, D., Wu, J., Dong, L.Y., Zhu, Y.J., and Hu, X. (2017). Flexible, high-wettability and fire-resistant separators based on hydroxyapatite nanowires for advanced lithium-ion batteries. Adv. Mater. *29*, 170354.

Mao, X., Shi, L., Zhang, H., Wang, Z., Zhu, J., Qiu, Z., Zhao, Y., Zhang, M., and Yuan, S. (2017). Polyethylene separator activated by hybrid coating improving Li^+ ion transference number and ionic conductivity for Li-metal battery. J. Power Sources *342*, 816-824.

Mihranyan, A., Llagostera, A.P., Karmhag, R., Strømme, M., and Ek, R. (2004). Moisture sorption by cellulose powders of varying crystallinity. Inter. J. Phar. *269*, 433-442.

Na, W., Lee, A.S., Lee, J.H., Hwang, S.S., Kim, E., Hong, S.M., and Koo, C.M. (2016). Lithium dendrite suppression with UV-curable polysilsesquioxane separator binders. ACS Appl. Mater. Interfaces *8*, 12852-12858.

Pan, L., Wang, H., Wu, C., Liao, C., and Li, L. (2015). Tannic-acid-coated polypropylene membrane as a separator for lithium-ion batteries. ACS Appl. Mater. Interfaces *7*, 16003-16010.

Peng, J.H., Bi, H.T., Lim, C.J., and Sokhansanj, S. (2013). Study on density, hardness, and moisture uptake of torrefied wood pellets. Energy Fuels *27*, 967-974.

Ryou, M.H., Lee, Y.M., Park, J.K., and Choi, J.W. (2011). Mussel-inspired polydopamine-treated polyethylene separators for high-power Li-ion batteries. Adv. Mater. *23*, 3066-3070.

Shi, C., Dai, J., Shen, X., Peng, L., Li, C., Wang, X., Zhang, P., and Zhao, J. (2016). A high-temperature stable ceramic-coated separator prepared with polyimide binder/Al_2O_3 particles for lithium-ion batteries. J. Membr. Sci. *517*, 91-99.

Wang, Z., Guo, F., Chen, C., Shi, L., Yuan, S., Sun, L., and Zhu, J. (2015). Self-assembly

of PEI/SiO_2 on polyethylene separators for Li-ion batteries with enhanced rate capability. ACS Appl. Mater. Interfaces *7*, 3314-3322.

Wang, Z., Pan, R., Ruan, C., Edström, K., Strømme, M., and Nyholm, L. (2017). Redox-active separators for lithium-ion batteries. Adv. Sci., 1700663.

Xu, W., Wang, Z., Shi, L., Ma, Y., Yuan, S., Sun, L., Zhao, Y., Zhang, M., and Zhu, J. (2015). Layer-by-layer deposition of organic-inorganic hybrid multilayer on microporous polyethylene separator to enhance the electrochemical performance of lithium-ion battery. ACS Appl. Mater. Interfaces *7*, 20678-20686.

Yang, P., Zhang, P., Shi, C., Chen, L., Dai, J., and Zhao, J. (2015). The functional separator coated with core-shell structured silica-poly(methyl methacrylate) sub-microspheres for lithium-ion batteries. J. Membr. Sci. *474*, 148-155.

Yang, Y., and Zhang, J. (2018). Highly stable lithium-sulfur batteries based on laponite nanosheet-coated celgard separators. Adv. Energy Mater. *8*, 1801778.

Zhang, J., and Seeger, S. (2011). Superoleophobic coatings with ultralow sliding angles based on silicone nanofilaments. Angew. Chem. Int. Ed. *50*, 6652-6656.

Zhang, J., Wang, A., and Seeger, S. (2014). *Nepenthes* pitcher inspired anti-wetting silicone nanofilaments coatings: preparation, unique anti-wetting and self-cleaning behaviors. Adv. Funct. Mater. *24*, 1074-1080.

Zhang, T.W., Shen, B., Yao, H.B., Ma, T., Lu, L.L., Zhou, F., and Yu, S.H. (2017). Prawn shell derived chitin nanofiber membranes as advanced sustainable separators for Li/Na-Ion batteries. Nano Lett. *17*, 4894-4901.

Zhu, X., Jiang, X., Ai, X., Yang, H., and Cao, Y. (2015). A highly thermostable ceramic-grafted microporous polyethylene separator for safer lithium-ion batteries. ACS Appl. Mater. Interfaces *7*, 24119-24126.

Zhu, X., Jiang, X., Ai, X., Yang, H., and Cao, Y. (2016). TiO_2 ceramic-grafted

polyethylene separators for enhanced thermostability and electrochemical performance of lithium-ion batteries. J. Membr. Sci. *504*, 97-103.
